# Lipidomics profile shows differences of polar lipids between donkey and bovine colostrum: a comparative study

**DOI:** 10.1016/j.fochx.2025.102798

**Published:** 2025-07-22

**Authors:** Yumeng Zhang, Jiali Chen, Zhenmin Liu, Shengyi Zhang, Jiale Wang, Junhua Shao, Xiqing Yue, Mohan Li

**Affiliations:** a College of Food Science, Shenyang Agricultural University, Shenyang 110866, China; b State Key Laboratory of Dairy Biotechnology, Shanghai Engineering Research Center of Dairy Biotechnology, Shanghai 200436, China; c School of Life Sciences & Biotechnology, Shanghai Jiao Tong University, Shanghai 200240, China

**Keywords:** Polar lipids, Donkey colostrum, Bovine colostrum, Lipidomics, Glycerophospholipid metabolism

## Abstract

Donkey colostrum (DC) is recognized as a valuable nutritional source; however, its extremely low lipid content (especially polar lipids) demands lipid supplementation during processing through the addition of fish oils, vegetable oils, or functional lipids. Therefore, a comprehensive characterization of polar lipids in both DC and bovine colostrum (BC) is required. In this study, totally 11 subclasses of 206 polar lipids in DC and BC were characterized. Using rigorous screening criteria (VIP > 1, *P* < 0.05, and fold change >2 or < 0.5), 141 lipid species were identified as having significantly different polar lipids (SDPLs) between DC and BC. Subsequent investigations revealed six key lipid metabolic pathways, with the glycerophospholipid metabolism being the most critical. These findings substantially enhance our understanding of the polar lipid differences between DC and BC, while providing a theoretical basis for synergistic utilization of the two milk sources to optimize nutritional enrichment strategies.

## Highlights

• 206 polar lipids of 11 subclasses were identified in donkey colostrum (DC) and bovine colostrum (BC).
• 141 significantly different polar lipids (SDPLs) were screened.
• These SDPLs were mainly enriched in the glycerophospholipid metabolism pathway.
• This study established the basis for synergistic BC and DC use to optimize nutritional enrichment.

## Graphical abstract

**Figure:**
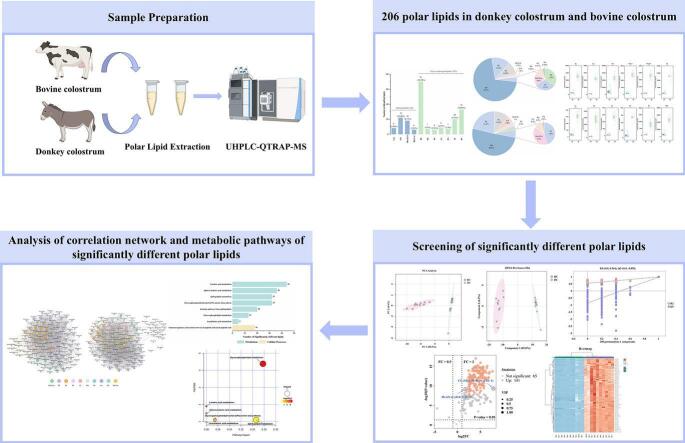

## 1 Introduction

Colostrum, the early milk secretion produced after parturition, is the main nutritional source for neonates in mammals (Brijesha & Aparna, 2017). Rich in fatty acids, immunoglobulins, and growth factors, it confers essential immune protection to neonates (Farkova et al., 2024). Bovine colostrum (BC), characterized by its rich lipid, vitamin, and lactoferrin contents, is widely utilized as a nutritional supplement for neonates, demonstrating therapeutic potential in alleviating gastrointestinal inflammation and respiratory disorders (Yalcintas et al., 2024). Notably, donkey colostrum (DC) has gained attention as a low-allergenicity human milk alternative owing to its similar whey protein to casein ratio to human milk and superior digestibility (M. Li et al., 2023). However, donkey milk (0.2%–1.8%) has lower fat content compared to bovine milk (3%–6%), which necessitates lipid fortification by supplementing donkey milk with fish oils, vegetable oils, or functional lipids (Bernard et al., 2025; Contarini, Pelizzola, Scurati, & Povolo, 2017; M. Li et al., 2022). High levels of specific lipids have been reported to positively affect intellectual development of children (Guxens et al., 2011). Therefore, comprehensive characterization of the lipid profiles in DC and BC is imperative to elucidate their differences.

Lipids are essential nutrients for energy transport, the construction of cellular architecture, and the delivery of bioactive molecules (J. Li et al., 2025). Milk lipids exist as fat globules encapsulated within a three-layer milk fat globule membrane (MFGM) abundant in polar lipids (Anto, Warykas, Torres-Gonzalez, & Blesso, 2020). Although polar lipids constitute less than 2% of the MFGM components, primarily glycerophospholipids (GPs), sphingolipids (SLs), and small amounts of cholesterol, they are vital to infant health and development (Jiang, Zhang, Yu, Wang, & Wei, 2025; Li et al., 2020). The main GPs found in milk include phosphatidylcholine (PC), phosphatidylethalomine (PE), phosphatidylinositol (PI), phosphatidylserine (PS), phosphatidylglycerol (PG), phosphatidic acid (PA), and cardiolipin (CL), while SLs mainly include sphingomyelin (SM), ceramide (Cer), dihexosylceramide (Hex2Cer), and hexosylceramide (HexCer) (Lopez, Briard-Bion, & Menard, 2014; Shuangshuang Wang et al., 2023). Previous studies have identified PC and PE as the predominant polar lipids in bovine milk, comprising 12%–48% and 3%–9% of the total polar lipids, respectively (Venkat, Chia, & Lambers, 2024). PC prevents lipid absorption disorders in infants, whereas PE modulates cell membrane fluidity (Z. Li & Vance, 2008; Vance, 2015). In contrast, donkey milk is characterized by relatively high levels of SM, which is a key component of myelination and neural plasticity in infants (Li et al., 2020). In addition, SM enhances digestive enzyme activity, thereby promoting intestinal maturation in neonates (Moloney et al., 2018). The polar lipid profile varies among mammals and is influenced by dietary environment, genetics, and physiological factors. To harness their unique nutritional advantages for infant dietary formulations, the differences in polar lipid composition and functionality of colostrum must be compared systematically.

As a critical component of metabolomics, lipidomics enables the multidimensional analysis of lipid molecules and their metabolic pathways (Y. Li et al., 2024). Mass spectrometry (MS) has been widely adopted in lipidomic studies owing to its unique advantages of high sensitivity, specificity, and capacity for microsample analysis (B. Wu et al., 2021). Milk lipids exhibit compositional complexity, with their profiles being affected by species, dietary and environmental factors (Lopez et al., 2014; Shang et al., 2022). Therefore, this technique provides an essential means for elucidating the differences in polar lipids from various milk sources. Recent studies have identified PE as the predominant phospholipid in donkey milk polar lipids (33.71%), with SM accounting for a slightly lower proportion (32.01%) (Jiang et al., 2022). In contrast, SM accounted for 25.89% of the total phospholipids in bovine milk, revealing variability across milk types. Recent evidence from UPLC-QTOF-MS analyses suggests neurodevelopment-associated SM differentiation between DC and mature donkey milk (Li, Li, Wu, et al., 2020). However, systematic comparisons of polar lipidomics between DC and BC are lacking.

In this study, a quantitative lipidomics approach based on ultra-high-performance liquid chromatography-quadrupole linear ion trap mass spectrometry (UHPLC-QTRAP-MS) was used to systematically characterize and quantify polar lipid profiles in DC and BC. Multivariate statistical analyses were performed to identify the significantly different polar lipids (SDPLs), followed by the construction of correlation network and potential metabolic pathways. This study establishes a fundamental framework for the targeted exploitation of the functionality of polar lipids in DC and BC. Furthermore, the findings not only guide polar lipid fortification strategies in infant formula but also advance the role of polar lipids in diversified nutritional interventions.

## 2 Materials and methods

### 2.1 Chemicals

The chemicals used in this study were sourced as follows: methanol, chloroform, acetone, acetonitrile (ACN), isopropanol (IPA), and ammonium acetate (all HPLC-grade) were purchased from CNW Technologies (Düsseldorf, Germany) and Merck (Darmstadt, Germany); Lipid standards were acquired from Avanti Polar Lipids (Alabaster, AL, USA).

### 2.2 Milk sample collection

DC samples were collected from 30 Chinese Dezhou donkeys (0–5 days postpartum) from a local farm in Dalian, China. All donkeys were healthy, aged 2–4 years, and foraged in the same pasture. Additionally, BC samples were obtained from 30 Chinese Holstein cows (1–5 days postpartum) at Huishan Farm in Shenyang, China. All cows were healthy and aged 2–4 years old. Immediately following collection, samples were aliquoted into sterile freezing tubes and transferred to −80 °C. For DC and BC samples, 30 samples each were randomly divided into 10 groups (3 parallel samples in each group), and 10 groups of both samples were analyzed. This study was approved by the Shenyang Agricultural University Animal Care and Use Ethics Committee (Permit No. SYXK <Liao> 2021–0010).

### 2.3 Polar lipid extraction

Milk lipids were extracted according to the method of Folch, Lees, and Sloane Stanley (1957). Briefly, 40 mL of chloroform/methanol solution was added to 10 mL of milk and centrifuged (4 °C, 30 min, 3500 ×g). The organic phase was carefully collected, and the aqueous phase was re-extracted by remixing with 20 mL of a chloroform/methanol solution. This re-extraction procedure was repeated twice. The collected organic phases were combined, washed with 0.9% NaCl, and blown dry under a stream of nitrogen to remove the solvent and obtain polar lipids. Finally, acetone was added to the lipids and stirred, and the polar lipid precipitate was collected by filtration (Jiang et al., 2022).

### 2.4 Polar lipid analysis

Lipidomic analysis of polar lipids in DC and BC was performed using an UHPLC system (AB Sciex, Framingham, MA, USA) equipped with a column (100 × 2.1 mm) and QTRAP (AB Sciex, Framingham, MA, USA). Mobile phase A was ACN/H_2_O (6:4, *v*/v) and mobile phase B was ACN/IPA (1:9, v/v), both of which contained 10 mM ammonium acetate. The mobile phase gradients were as follows: 0 min, 20% B; 2 min, 30% B; 4 min, 60% B; 9 min, 85% B; 14 min, 90% B, 15.5 min, 95% B; and 17.5 min, 20% B. ESI source conditions were as follows: ion source gas 1, 0.31 MPa; ion source gas 2, 0.38 MPa; curtain gas, 0.24 MPa; source temperature, 500 °C; 5500 or − 4500 V in positive and negative modes, respectively. Multiple reaction monitoring (MRM) experiments were performed to obtain QQQ scans, and the collision gas (nitrogen) was set to 0.034 MPa.

### 2.5 Data processing and annotation

For lipid identification, peak extraction was performed using Lipid Analyzer (Tu, Yin, Xu, Wang, & Zhu, 2018). Raw data files were converted to the mzXML format to ensure compatibility with the Lipid Analyzer platform. MS1 peak data (*m*/*z*, retention time (RT), and peak area) were acquired using XCMS software and used to extract the MS/MS spectra. The lipids were identified by matching the spectra to an in-house MS/MS spectral library. Additionally, lipid quantification was achieved using the peak area, stable isotope-labeled internal standards, and relevant fragment information.

### 2.6 Statistical analysis

Principal component analysis (PCA), orthogonal partial least squares discriminant analysis (OPLS-DA), hierarchical cluster analysis, and lipid metabolic pathways were conducted using MetaboAnalyst (http://www.metaboanalyst.ca). Univariate analyses following Student's *t*-tests (*p*-value), VIP values, and fold change (FC) were performed. FC represents mean DC content/ mean BC content. Volcano plots were generated using the GraphPad Prism 10.1.2 software (GraphPad Software Inc., La Jolla, CA, USA). Spearman correlation networks for SDPLs were constructed using R packages and visualized in Cytoscape 3.10.1 (Seattle, USA). This was mainly based on the Kyoto Encyclopedia of Genes and Genomes (KEGG) pathway database (https://www.kegg.jp/kegg/), the Human Metabolome Database (HMDB, http://www.hmdb.ca), the Reactome database (https://curator.reactome.org/), and the WikiPathways database (https://www.wikipathways.org/).

## 3 Results

### 3.1 Qualitative analysis of DC and BC polar lipids

**Fig. 1:**
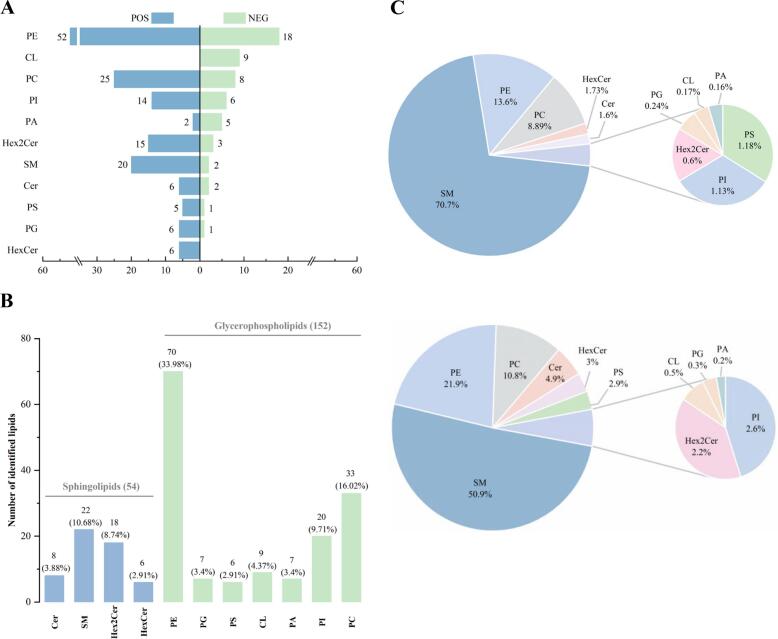
The identified lipid numbers between positive ion modes (POS) and negative ion modes (NEG) (A). Number of lipid species identified in 11 lipid subclasses (B). Percentage composition of the content of each lipid subclass in DC or BC (C). DC = donkey colostrum; BC = bovine colostrum. PC, phosphatidylcholine; PE, phosphatidylethalomine; PI, phosphatidylinositol; PS, phosphatidylserine; PG, phosphatidylglycerol; PA, phosphatidic acid; CL, cardiolipin; SM, sphingomyelin; Cer, ceramide; Hex2Cer, dihexosylceramide; HexCer, hexosylceramide.

In this study, lipid profiling was conducted on 20 groups of samples (10 DC and 10 BCE) in both positive and negative ion modes. Data were normalized using the total ion current method, and the results are shown in Fig. 1A. Following rigorous filtering to remove the lipids detected in both ionization modes, 206 polar lipids were identified across the DC and BC samples (Fig. 1B and Table S1). The identified lipids were classified into 11 subclasses, including 70 PE (33.98%), 33 PC (16.02%), 22 SM (10.68%), 20 PI (9.71%), 18 Hex2Cer (8.74%), 9 CL (4.37%), 8 Cer (3.88%), 7 PA (3.4%), 7 PG (3.4%), 6 HexCer (2.91%), and 6 PS (2.91%). Subsequently, all polar lipids were grouped into two major categories: 54 SLs (26.21%) and 152 GPs (73.79%).

### 3.2 Qualitative analysis of DC and BC polar lipids

**Fig. 2:**
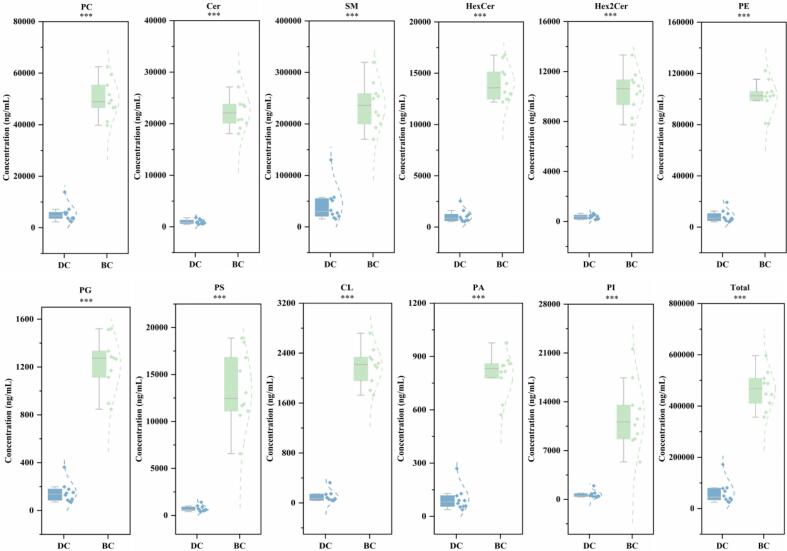
Comparison of each lipid subclass between DC and BC, the Y-axis indicates the concentration of lipids (ng/mL) (*w*/w). DC = donkey colostrum; BC = bovine colostrum. PC, phosphatidylcholine; PE, phosphatidylethalomine; PI, phosphatidylinositol; PS, phosphatidylserine; PG, phosphatidylglycerol; PA, phosphatidic acid; CL, cardiolipin; SM, sphingomyelin; Cer, ceramide; Hex2Cer, dihexosylceramide; HexCer, hexosylceramide. *** means *P* < 0.001.

As shown in Fig. 1C, SM constituted the most abundant subclass, accounting for 70.7% of the DC and 50.9% of the BC polar lipids, followed by PE (DC 13.6% and 21.9 BC%) and PC (DC 8.89% and 10.8 BC%). The contents of the 11 lipid subclasses are represented as boxplots in Fig. 2. Notably, BC displayed significantly higher concentrations across all 11 subclasses than DC (*P* < 0.001). Among the 206 identified polar lipids, only seven showed higher concentrations in DC than in BC, namely PC(P-20:0/20:1), SM(d14:1/14:0), SM(d14:0/14:0), SM(d14:0/26:1), SM(d17:0/26:1), PE(18:2/0:0), and Hex2Cer(d14:0/18:0) (Fig. S1.).

### 3.3 Lipid pattern recognition analysis of DC and BC polar lipids

**Fig. 3:**
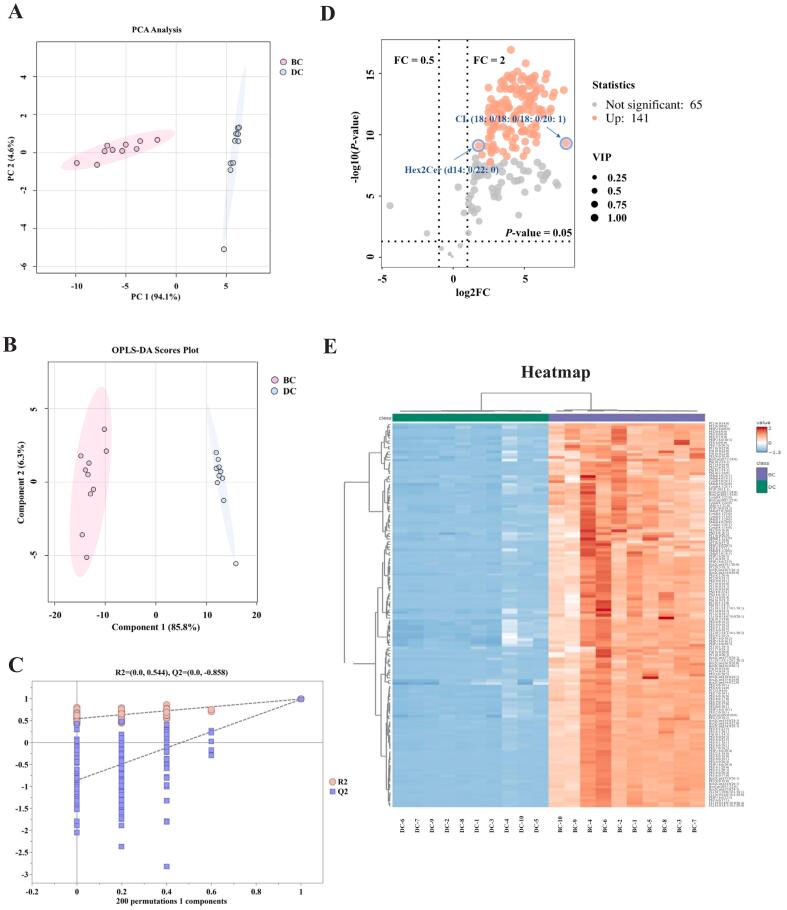
Plot of principal component analysis (PCA) scores (A), orthogonal partial least squares discriminant analysis (OPLS-DA) score plot (B), corresponding OPLS-DA validation (C) and volcano plot (D) for DC and BC. Heatmap analysis of 141 SDPLs between DC and BC (E). DC = donkey colostrum; BC = bovine colostrum; SDPLs: significantly different polar lipids; FC: fold change; VIP: variable importance in projection.

To visualize inter- and intra-group variations in polar lipid profiles between DC and BC, multivariate analyses were conducted using PCA and OPLS-DA in the SIMCA software. The first two principal components of PCA — PC1 and PC2 — were extracted from the PCA scores, accounting for 94.1% and 4.6% of the total variance (cumulative 98.6%), respectively (Fig. 3A). The PCA score plots show a clear separation tendency between the DC and BC groups, indicating a significant difference between them. Moreover, OPLS-DA further enhances model specificity, thereby improving analytical accuracy (Shaolei Wang et al., 2025). As shown in Fig. 3B, OPLS-DA enhanced the separation of the DC and BC groups, indicating the presence of different lipid compositions in the two samples. Model validity was verified using seven cross-validation tests and 200 random permutation tests (Fig. 3C). The R^2^ and Q^2^ regression lines decreased with decreasing replacement retention. The intersection of the Q^2^ regression line with the vertical axis was less than 0 (Q^2^ = −0.858), showing a well-fitted model.

### 3.4 Screening for SDPLs between DC and BC

In this study, OPLS-DA model projections (VIP > 1), Student's *t*-tests (*P* < 0.05), and fold change (FC > 2 or FC < 0.5) were applied as screening criteria to analyze the differences in polar lipid composition between DC and BC. Among these, 141 SDPLs were identified, including 8 Cer, 10 SM, 15 Hex2Cer, 6 HexCer, 57 PE, 5 PG, 3 PS, 8 CL, 6 PA, and 23 PC. Table S2 provides detailed information on these 141 polar lipids, showing significant differences between the DC and BC groups. Of the SDPLs, CL (18:0/18:0/18:0/20:1) exhibited the highest FC value (FC = 224.855), whereas Hex2Cer (d14:0/22:0) displayed the lowest FC value (FC = 3.4735). Subsequently, differences in the DC and BC polar lipid levels were visualized on volcano plots, confirming the significant upregulation of all 141 SDPLs (Fig. 3D). As shown in Fig. 3E, the heatmap of SDPLs visually demonstrated the difference in expression abundance between DC and BC. The findings showed that the lipid levels of all the SDPLs in the BC sample classes were significantly higher than those in the DC group.

### 3.5 Correlation network analysis of SDPLs between DC and BC

**Fig. 4:**
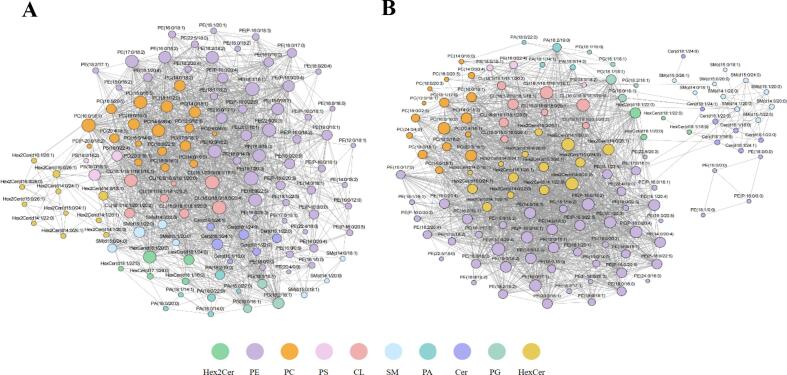
Spearman's correlation network (*p* < 1e-6) of 141 SDPLs in DC (A) and BC (B). Lipid species were color-coded into ten major lipid subclasses. The size of the circle represents the level of content. DC = donkey colostrum; BC = bovine colostrum; SDPLs: significantly different polar lipids. PC, phosphatidylcholine; PE, phosphatidylethalomine; PI, phosphatidylinositol; PS, phosphatidylserine; PG, phosphatidylglycerol; PA, phosphatidic acid; CL, cardiolipin; SM, sphingomyelin; Cer, ceramide; Hex2Cer, dihexosylceramide; HexCer, hexosylceramide.

The synergistic function and holistic regulation of lipids, as molecules underlying life activities, are essential for maintaining homeostasis in organisms. The correlation between various lipids was revealed using an unweighted correlation network analysis of 141 SDPLs. As illustrated in Fig. 4A and B, the majority of the lipids were highly correlated. Lipids belonging to the same lipid subclasses form densely interconnected clusters. Among them, 1398 correlations were identified in the DC group compared to 1525 correlations in the BC group (*P* < 1e-6).

### 3.6 Differentially expressed lipid metabolic pathways

**Fig. 5:**
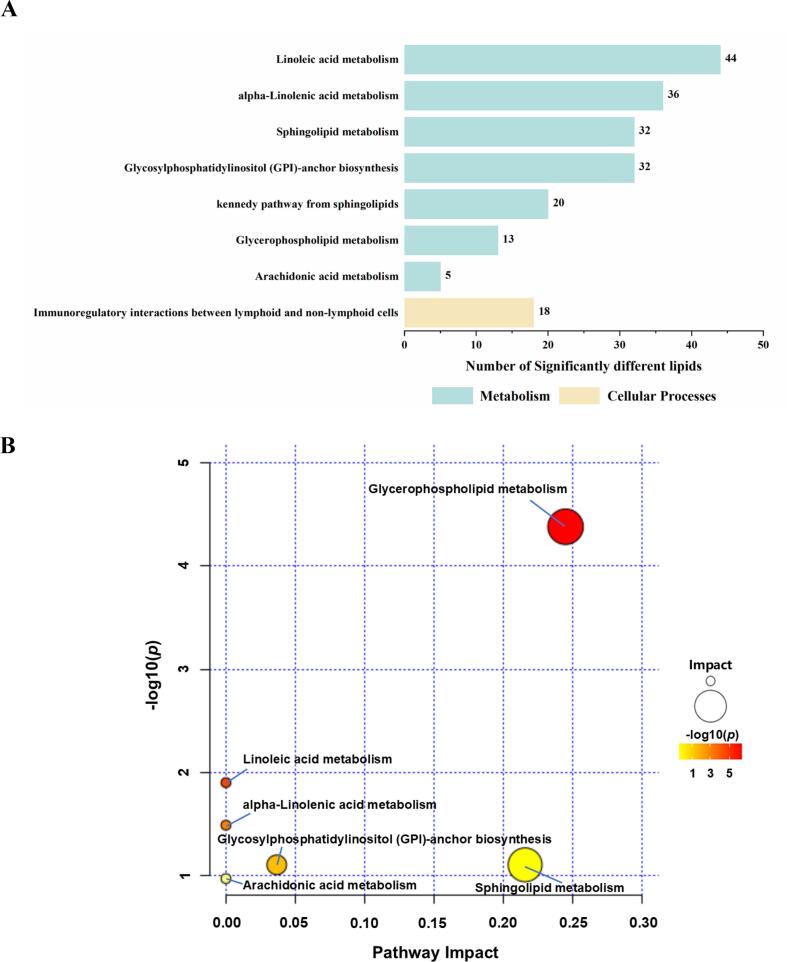
KEGG pathway analysis of SDPLs between DC and BC (A). Metabolomic profiles of significantly different polar lipid metabolic pathways in DC and BC (B). DC = donkey colostrum; BC = bovine colostrum; SDPLs: significantly different polar lipids.

Eight lipid metabolic pathways were identified by mapping the 141 SDPLs to integrated databases (KEGG, HMDB, Reactome, and WikiPathways) (Fig. 5A). Subsequently, the lipid classes and contents of these SDPLs were introduced into MetaboAnalyst 6.0 to obtain the six most significantly related metabolic pathways (Fig. 5B and Table S3). GP metabolism was the most prominent pathway, followed by linoleic acid metabolism, alpha-linolenic acid metabolism, glycosylphosphatidylinositol (GPI)-anchor biosynthesis, SL metabolism, and arachidonic acid metabolism.

## 4 Discussion

Colostrum, a concentrated source of nutrients, is an optimal nutritional source for neonates. Among the diverse nutritional components, lipids are involved in fundamental physiological processes, including cellular membrane formation (Kanmaz et al., 2025). Notably, although polar lipids constitute a relatively minor proportion of the total lipid content in the colostrum, they exert significant beneficial effects on neonatal immune protection and human health. Consequently, comprehensive characterization of polar lipids in donkey and bovine milk is imperative.

Our comprehensive analysis identified 206 polar lipids across 11 subclasses in DC and BC, comprising 8 Cer (3.88%), 22 SM (10.68%), 18 Hex2Cer (8.74%), 6 HexCer (2.91%), 70 PE (33.98%), 7 PG (3.4%), 6 PS (2.91%), 9 CL (4.37%), 7 PA (3.4%), 20 PI (9.71%), and 33 PC (16.02%). Notably, SM, a key component of MFGM, represented the richest polar lipid in both DC and BC samples. Studies have found that ceramide produced by SM hydrolysis induces immune response by modulating PC12 cell differentiation (Kagan, Stoyanova, Lockshin, & Zakeri, 2022). Furthermore, SM offers an essential lipid backbone for myelination, facilitating rapid signal transduction in the central nervous system, thereby affecting neonatal neurodevelopment. Notably, SM constituted a higher proportion in DC than in BC. However, currently, there is limited understanding of the differences in the functions performed by SM in ruminants (bovine) and non-ruminants (donkey). Therefore, it would be meaningful to further explore whether non-ruminants (donkey) neurodevelopment has a high nutritional requirement for SM. In addition, the top three lipid subclasses dominant in BC were SM (50.9%), PE (21.9%), and PC (10.8%). This distribution pattern is similar to that reported in previous studies on polar lipids in bovine milk (Gu et al., 2024; Z. Liu, Logan, Cocks, & Rochfort, 2017).

Subsequently, a comparative analysis of total polar lipids and lipid subclass levels was conducted between the DC and BC. BC had a higher total polar lipid level than DC, and the contents of all 11 lipid subclasses were higher in BC (*P* < 0.05). This observation aligns with previous reports documenting lower lipid content in donkey milk than in bovine milk, where most polar lipids exhibit positive correlations with total fat content (K. Liu et al., 2024). This can be attributed to the fact that ruminants generally have higher lipid levels than non-ruminants. Ruminants (bovine) mediate fatty acid conversion through rumen microbes, whereas non-ruminants (donkey) do not need to rely on such complex microbial systems for lipid catabolism and absorption (Y. Wu et al., 2023). Interestingly, non-ruminant milk possesses MFGM characteristics that are more conducive to human absorption than ruminant milk. The phenomenon may be explained by the much smaller fat globules present in donkey milk, exhibiting a mean size of 1.5 ± 0.15 μm, considerably lower than in other species (bovine 4.96  ± 0.74 μm) (Wei et al., 2022). Indeed, changes in milk fat concentration and yield are correlated with milk fat globule size because of the specific synthesis of membrane components and the impact of lipogenic pathways. Notably, the lipid digestion efficiency and metabolic outcomes in infants are influenced by fat globule size, suggesting DC as a superior source of nutrients in terms of polar lipids (Lazarevic et al., 2023). This further demonstrates the differences in polar lipids in the colostrum of ruminants and non-ruminants, providing possibilities for the development of dairy-based food systems that can synergistically utilize different milk sources.

To comprehensively elucidate the differences in polar lipid composition between ruminants (bovine) and non-ruminants (donkey), 141 SDPLs were identified between DC and BC based on quantitative lipidomics. The major SDPLs comprised 8 Cer, 10 SM, 15 Hex2Cer, 6 HexCer, 57 PE, 5 PG, 3 PS, 8 CL, 6 PA, and 23 PC. Notably, PE and PC were the predominant SDPLs in DC and BC. PE participates in myelin sheath formation and modulates the neural signal transmission rate to affect brain development, while concurrently regulating immune responses through TLR4-mediated signaling pathways (Calzada, Onguka, & Claypool, 2016). Among them, the significant difference observed in PC (18:0/18:1) may be related to developmental differences in brain function and cognitive abilities between donkey and bovine (Yang, Lv, Zhang, Wang, & Deng, 2024). In addition, Hex2Cer (d14:1/20:0) exhibited significant differences in DC and BC, which may be influenced by species disparities or dietary factors. Hex2Cer, formed by the two-step glycosylation of Cer, acts as a lipid regulator of cell differentiation and apoptosis (Jiang et al., 2025). More importantly, we found that CL (18:0/18:0/18:0/20:1) exhibited the largest FC value (FC = 224.855) among these SDPLs. As a key component of the inner mitochondrial membrane of mammals, the conical molecular structure of CL tends to form structures with greater membrane curvature (Corey, Harrison, Stansfeld, Sansom, & Duncan, 2022; Piccinini, Kohlbrecher, Moussaoui, Winter, & Prevost, 2024). This implies the formation of inner mitochondrial cristae in the intestinal tract or immune cells of neonates, which accelerates energy metabolism. Overall, the significant differences in polar lipids between DC and BC provide a viable opportunity to optimize infant nutrition and dairy processing. The high SM content of DC can be synergistically combined with the PC and PE of BC to enhance neurodevelopmental support and intestinal barrier integrity. This precise fortification strategy provides a viable approach for developing formulas tailored to preterm infants. On the other hand, the extremely high abundance of CL in BC, especially CL (18:0/18:0/18:0/20:1), enhances mitochondrial energy metabolism efficiency. Supplementation with CL represents a promising strategy for improving cardiovascular health.

A similar trend for SDPLs in the same subclass supports the correlation between lipid levels. These SDPLs showed a high correlation in both DC and BC, with correlations being stronger for the same subclass of polar lipids than for different subclassses. In comparison, the DC group exhibited tighter clusters, reflecting a strong association of polar lipids in donkey milk. Notably, the node network constituted by SM exhibited differences across different milk sources, which is consistent with previous results. Moreover, Cer was found to exhibit strong inter-group association with other SDPLs in DC, but weak correlation in BC. As a central regulator of cellular differentiation and apoptosis, Cer serves as a critical node in intra- and intercellular lipid signaling (Pant, Aguilera-Albesa, & Pujol, 2020). Collectively, our findings emphasize a not negligible role of polar lipids in lipid anabolism in the colostrum of different species.

The present study not only identified SDPLs by comprehensive lipidomics of DC and BC, but also elucidated their involvement in critical lipid metabolic pathways. Among the six most significantly associated pathways identified, GP metabolism emerged as the most pivotal in both colostrum types. This may be attributed to the significant differences in PE and PC levels, which aligned with our distinct lipid profiles. Specifically, BC exhibited significantly higher PE and PC concentrations than DC (*P* < 0.05), directly modulating the GP metabolic flux. Elevated levels of PE and PC enhance membrane fluidity to facilitate vesicle formation, thereby influencing intercellular communication and nutrient transport, while simultaneously playing indispensable roles in intestinal maturation and neural development in newborns. Additionally, the significantly different pathways encompassed SL metabolism. The differences in the significant metabolic pathways in SL may be closely related to milk fat globule size. Donkey milk contains smaller fat globules than ruminant milk, and a higher proportion of SM is associated with membrane stability (Thum, Roy, Everett, & McNabb, 2023). Overall, our findings provide insights into the differences in the lipid metabolic pathways involved in polar lipids in the colostrum of the two mammals. Considering the increasing interest in adding polar lipid fractions to food systems, pathway analysis offers the potential for translational applications of mammalian colostrum.

## 5 Conclusion

In this study, UHPLC-QTRAP-MS-based lipidomic analysis of polar lipids in donkey and bovine colostrum was performed. In summary, 206 polar lipids from 11 lipid subclasses were identified in DC and BC, and 141 SDPLs were screened. Moreover, the correlation networks and associated metabolic pathways of these SDPLs revealed the prominence of GP metabolism among the eight differential pathways. This study advances the development of the comparative polar lipidomics of colostrum, thereby providing a theoretical basis for optimizing bovine milk processing. Future investigations should focus on the synergistic utilization of different species of milk sources to address nutritional requirements for both neonates and adults, as well as provide targeted supplementation to support development and health.

## CRediT authorship contribution statement

**Yumeng Zhang:** Writing – original draft, Methodology, Formal analysis, Conceptualization. **Jiali Chen:** Writing – original draft, Formal analysis, Data curation. **Zhenmin Liu:** Data curation. **Shengyi Zhang:** Formal analysis. **Jiale Wang:** Data curation. **Junhua Shao:** Writing – review & editing, Visualization. **Xiqing Yue:** Writing – review & editing, Project administration, Funding acquisition. **Mohan Li:** Writing – review & editing, Visualization, Supervision, Project administration.

## Declaration of competing interest

The authors declare that they have no known competing financial interests or personal relationships that could have appeared to influence the work reported in this paper.

## Data Availability

Data will be made available on request.

## Appendix A Supplementary data

Supplementary material 1 Image 1

Supplementary material 2 Image 2
