# Supplementary material for: Lipidomics profile shows differences of polar lipids between donkey and bovine colostrum: a comparative study
Source: Food Chem X. 2025 Jul 22;29:102798. doi: 10.1016/j.fochx.2025.102798 (PMC12311604; doi:10.1016/j.fochx.2025.102798)
Supplement: Supplementary file 1 — Supplementary material 1 [file mmc1.docx]

Figure captions:

Figure. S1. Quantification of polar lipids in DC and BC. Quantification of glycerophospholipids (A). Quantification of sphingolipids (B). SM, sphingomyelin; Cer, ceramide; Hex2Cer, dihexosylceramide; HexCer, hexosylceramide; PC, phosphatidylcholine; PE, phosphatidylethalomine; PI, phosphatidylinositol; PS, phosphatidylserine; PG, phosphatidylglycerol; PA, phosphatidic acid; CL, cardiolipin.


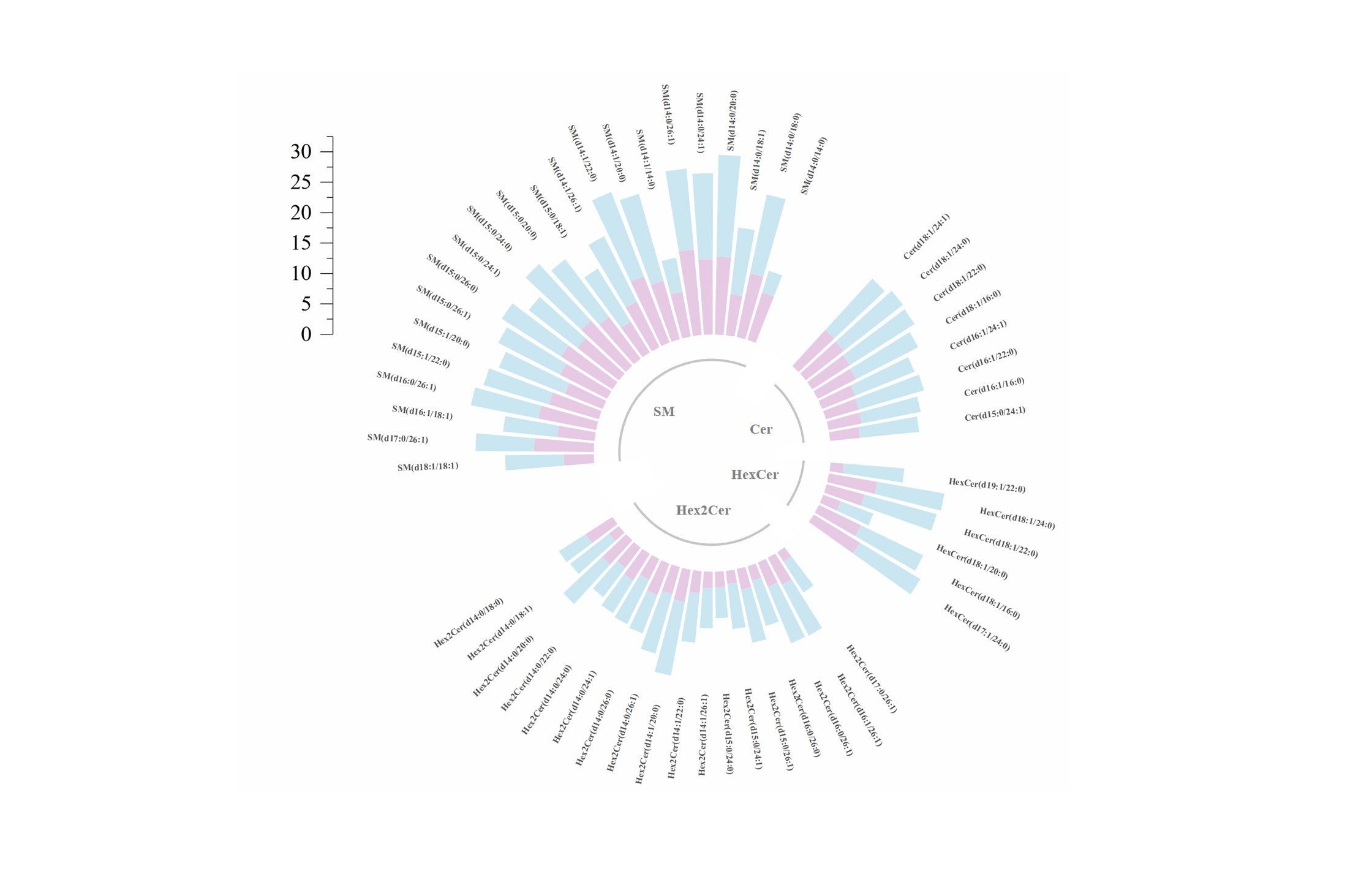


Figure. S1. Quantification of polar lipids in DC and BC. Quantification of glycerophospholipids (A). SM, sphingomyelin; Cer, ceramide; Hex2Cer, dihexosylceramide; HexCer, hexosylceramide.


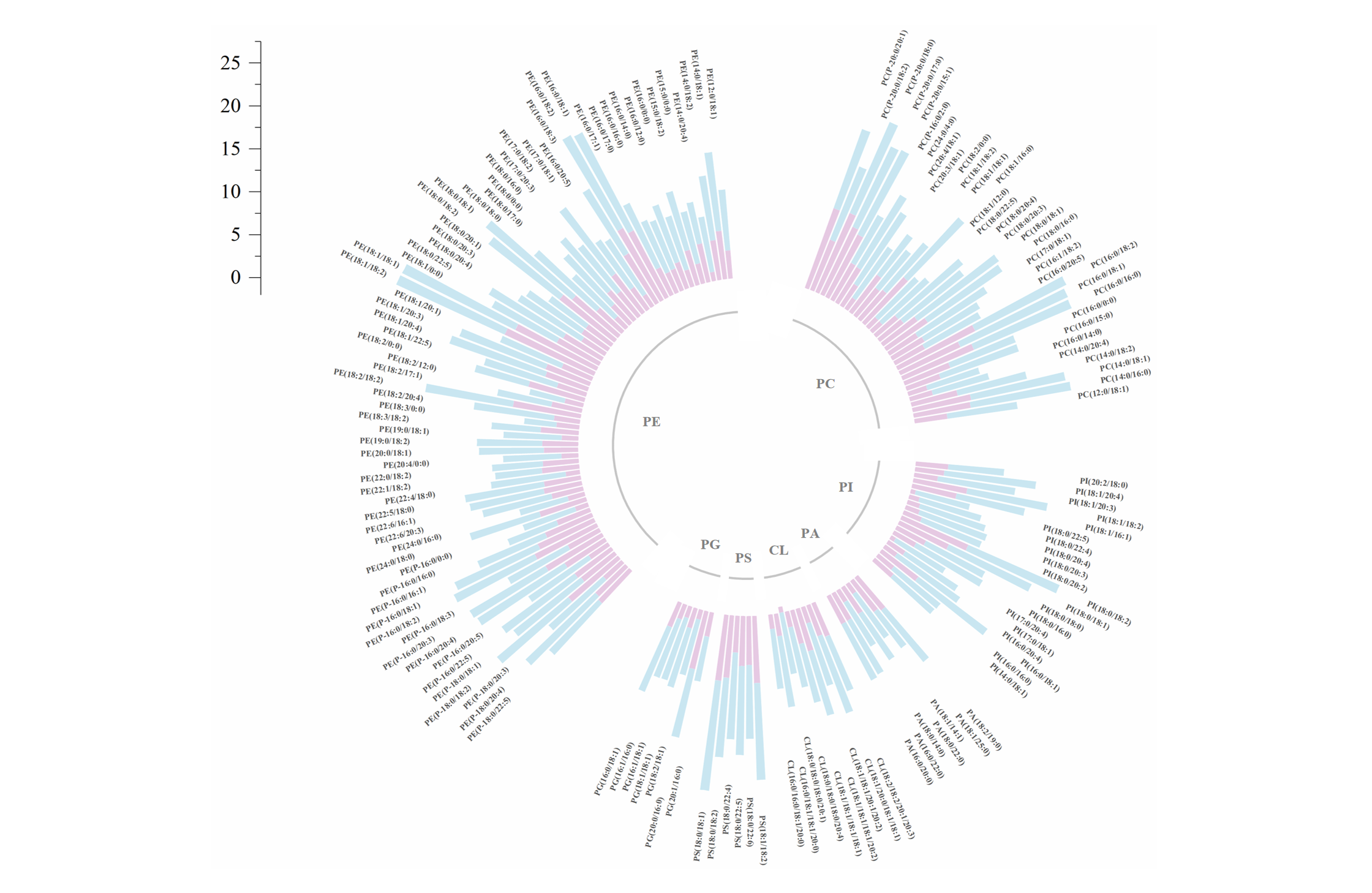


Figure. S1. Quantification of polar lipids in DC and BC. Quantification of sphingolipids (B). PC, phosphatidylcholine; PE, phosphatidylethalomine; PI, phosphatidylinositol; PS, phosphatidylserine; PG, phosphatidylglycerol; PA, phosphatidic acid; CL, cardiolipin.
