# Supplementary material for: Lipidomics profile shows differences of polar lipids between donkey and bovine colostrum: a comparative study
Source: Food Chem X. 2025 Jul 22;29:102798. doi: 10.1016/j.fochx.2025.102798 (PMC12311604; doi:10.1016/j.fochx.2025.102798)
Supplement: Supplementary file 2 — Supplementary material 2 [file mmc2.docx]

Table captions:

Table S1. Polar lipids contents in donkey colostrum (DC) and bovine colostrum (BC) (ng/mL).

Table S2. Identification of significantly different polar lipids between donkey colostrum and bovine colostrum.

Table S3. Metabolic pathway identified from the significantly different polar lipids between donkey colostrum and bovine colostrum.

Table S1. Polar lipids contents in donkey colostrum (DC) and bovine colostrum (BC) (ng/mL).

| id | MS2 name | mzmed | rtmed | DC-1 | DC-2 | DC-3 | DC-4 | DC-5 | DC-6 | DC-7 | DC-8 | DC-9 | DC-10 | BC-1 | BC-2 | BC-3 | BC-4 | BC-5 | BC-6 | BC-7 | BC-8 | BC-9 | BC-10 |
| --- | --- | --- | --- | --- | --- | --- | --- | --- | --- | --- | --- | --- | --- | --- | --- | --- | --- | --- | --- | --- | --- | --- | --- |
| POS | PC(16:0/0:0) | 496.3342889 | 86.524 | 66.40553525 | 40.17849037 | 34.54456162 | 32.31467888 | 98.75841985 | 61.94681945 | 28.3628127 | 37.53243472 | 74.29124358 | 41.27905497 | 272.9826902 | 254.9884293 | 331.807733 | 292.7866597 | 290.5668918 | 253.4168345 | 279.030439 | 300.6236458 | 314.3200938 | 290.9833102 |
| POS | PC(18:2/0:0) | 520.3338672 | 72.844 | 44.70970242 | 25.7921187 | 26.80311848 | 21.71345267 | 115.1055426 | 71.74604225 | 23.21246658 | 24.85412538 | 41.32908817 | 27.71267688 | 50.3425773 | 44.93666368 | 88.66823207 | 58.92546957 | 63.64389652 | 49.53952965 | 47.30324542 | 77.8397009 | 54.75088142 | 60.00432493 |
| POS | PC(P-16:0/2:0) | 522.3500164 | 88.6025 | 26.41397808 | 25.8215142 | 24.12805522 | 17.37557148 | 51.65117068 | 49.89256687 | 17.69779825 | 25.15997243 | 39.790858 | 26.26231987 | 71.01698928 | 106.439786 | 80.95743678 | 69.40727895 | 117.7769991 | 86.33098067 | 95.82720753 | 60.15615863 | 76.17860562 | 65.89117662 |
| POS | Cer(d16:1/16:0) | 510.4835111 | 306.534 | 60.32603723 | 47.22602347 | 43.94409085 | 40.54762948 | 53.94358642 | 52.87373325 | 42.50058258 | 47.15894422 | 48.3770467 | 54.27653708 | 682.4415402 | 941.3704113 | 1069.462762 | 772.1140597 | 1178.730148 | 997.0070375 | 1044.316985 | 697.7619052 | 856.3048947 | 771.8175695 |
| POS | Cer(d18:1/16:0) | 538.5152762 | 354.718 | 101.3734509 | 130.7298269 | 112.3988309 | 111.8548983 | 177.6195145 | 159.0518318 | 152.2985284 | 95.81528303 | 203.5033898 | 129.4057703 | 1989.474463 | 3482.072272 | 3112.05227 | 2496.838032 | 3912.310183 | 2831.09687 | 3434.748835 | 2209.970595 | 2558.873747 | 2067.101308 |
| POS | Cer(d16:1/22:0) | 594.5775694 | 439.503 | 94.31336078 | 46.41907508 | 53.24912163 | 24.23824705 | 67.0547523 | 70.01775643 | 31.6503854 | 35.08954148 | 49.00052642 | 42.83690097 | 1735.599265 | 2541.153755 | 2881.755355 | 2078.654768 | 3183.14516 | 2373.64104 | 2472.497097 | 1728.396058 | 2007.002943 | 1869.49117 |
| POS | Cer(d18:1/22:0) | 622.6069902 | 474.7575 | 319.4166223 | 165.306803 | 165.6763203 | 75.9506123 | 200.8633677 | 250.16892 | 117.8070907 | 90.60886013 | 129.7757108 | 117.2999312 | 5992.758008 | 6300.954558 | 7680.581198 | 5681.227367 | 8150.51212 | 6797.981047 | 6310.799523 | 4935.267983 | 6016.667223 | 5705.532973 |
| POS | Cer(d18:1/24:1) | 648.6236529 | 474.1585 | 510.6631005 | 196.2081685 | 281.9760465 | 81.99390845 | 627.8073288 | 296.6884565 | 119.9974011 | 104.8475626 | 213.0950583 | 163.8863576 | 2403.686183 | 2674.362685 | 3147.60468 | 2384.773393 | 3565.298243 | 2774.257518 | 2446.298445 | 2379.107025 | 2354.224372 | 2296.939662 |
| POS | Cer(d18:1/24:0) | 650.6394814 | 505.753 | 427.677724 | 186.6148968 | 237.084549 | 116.6709569 | 361.5840768 | 342.4860262 | 159.9972021 | 114.8052128 | 217.1940423 | 167.894841 | 5669.534627 | 5101.926718 | 6556.772872 | 5263.668253 | 7078.673967 | 5178.96044 | 5108.408817 | 4370.30052 | 4122.066957 | 4646.086442 |
| POS | SM(d14:1/14:0) | 619.475411 | 160.5525 | 279.5346093 | 56.3333902 | 62.394258 | 101.7928175 | 542.5637167 | 136.1226069 | 74.55286575 | 172.8234318 | 216.020231 | 171.8592665 | 49.96707173 | 59.84075365 | 52.78316782 | 39.11059275 | 72.71754068 | 55.4851709 | 54.88607553 | 44.96877408 | 41.09213233 | 34.82402045 |
| POS | SM(d14:0/14:0) | 621.4904127 | 175.858 | 465.9412985 | 97.62021825 | 143.3153202 | 178.2425943 | 550.0159353 | 163.305657 | 124.3368767 | 322.2699137 | 460.6360832 | 342.1858285 | 11.3249011 | 15.85815137 | 15.63400809 | 11.33194323 | 17.45203972 | 13.87925746 | 13.98677994 | 10.05051714 | 12.01922794 | 9.607617568 |
| POS | SM(d14:0/18:1) | 675.5397719 | 261.557 | 201.2467825 | 67.0433609 | 65.87062328 | 55.29272833 | 263.5388288 | 86.07270652 | 53.60306355 | 100.5081634 | 121.8679275 | 115.4694597 | 2177.42663 | 2219.250043 | 2635.549467 | 1976.780115 | 2832.797955 | 2098.977865 | 2360.996187 | 1321.513415 | 1779.97607 | 1129.069203 |
| POS | SM(d14:0/18:0) | 677.5501422 | 398.2435 | 1999.218962 | 852.9897192 | 1604.692587 | 920.9887485 | 6827.969477 | 2070.572147 | 453.9719273 | 498.3939713 | 853.9881652 | 717.0916947 | 5909.264988 | 10387.75088 | 11138.02031 | 8887.272497 | 12928.27032 | 10260.08859 | 10186.71702 | 7122.994925 | 8450.032068 | 8067.839757 |
| POS | SM(d15:0/18:1) | 689.5551345 | 285.969 | 76.82077113 | 44.11045273 | 39.8640711 | 33.98965932 | 137.3675906 | 57.54565367 | 35.50261635 | 52.87403043 | 120.4678396 | 52.92769653 | 975.861348 | 1485.900202 | 1026.381171 | 831.1203472 | 1668.546402 | 1310.698164 | 1457.176314 | 1041.572681 | 1037.000101 | 896.283848 |
| POS | SM(d16:1/18:1) | 701.5576157 | 276.919 | 81.43810815 | 79.46784703 | 56.79064918 | 61.58668038 | 93.94540025 | 78.70288037 | 66.37916392 | 61.8060395 | 64.50100137 | 66.74932488 | 418.729525 | 505.5187848 | 556.4491252 | 426.2449802 | 842.4488147 | 585.9212143 | 545.8156403 | 377.4676535 | 476.3123277 | 285.3831478 |
| POS | SM(d14:1/20:0) | 703.571219 | 317.7295 | 1692.803312 | 751.6896292 | 677.7117515 | 392.3843278 | 2344.182772 | 700.3460337 | 553.6061038 | 723.5097478 | 992.812004 | 851.4233707 | 21999.41125 | 41242.83087 | 26572.2377 | 22819.631 | 42728.62057 | 30261.37113 | 39939.32198 | 24103.40403 | 28561.52963 | 18049.85797 |
| POS | SM(d14:0/20:0) | 705.5828309 | 435.422 | 10617.88656 | 5891.395458 | 8432.756078 | 3680.993132 | 16960.32337 | 11511.36568 | 3578.062492 | 2488.397002 | 4783.079372 | 3423.55438 | 70048.03412 | 110027.9851 | 130162.1648 | 105428.9214 | 145613.9234 | 121335.6704 | 113600.2153 | 85167.09045 | 99402.45705 | 93842.90345 |
| POS | SM(d15:1/20:0) | 717.581365 | 434.286 | 165.9936095 | 100.0215348 | 89.60269205 | 63.3686353 | 122.6409526 | 157.9935821 | 85.009618 | 56.31960957 | 119.7556386 | 73.40752182 | 2042.180442 | 3242.566825 | 4013.952565 | 3088.920745 | 4491.76942 | 3605.34124 | 3419.151862 | 2787.610937 | 2943.762583 | 2633.923587 |
| POS | SM(d15:0/20:0) | 719.5958285 | 463.78 | 695.5010758 | 234.9849838 | 231.0574402 | 248.4038027 | 451.8620595 | 761.9296737 | 314.0279477 | 283.092466 | 584.593465 | 198.4656153 | 3376.839325 | 3503.509278 | 4401.821405 | 3298.89487 | 5017.864518 | 4234.10236 | 3588.926858 | 3439.143382 | 4162.041105 | 2941.92202 |
| POS | SM(d18:1/18:1) | 729.5884409 | 326.572 | 43.31427423 | 27.54786577 | 24.06825105 | 21.47056503 | 66.02331215 | 35.39141498 | 18.54313672 | 24.08278305 | 26.35722563 | 24.0811019 | 592.0910067 | 1036.916082 | 575.8123177 | 651.8418028 | 1266.064591 | 811.7819737 | 1146.488615 | 682.1237983 | 714.5377827 | 511.6872747 |
| POS | SM(d14:1/22:0) | 731.5966206 | 451.77 | 5792.958977 | 3271.591928 | 2435.982018 | 2179.462767 | 2358.004575 | 5120.565582 | 3259.659245 | 2190.219972 | 4458.573052 | 2527.404568 | 22328.02638 | 28391.03423 | 35285.3468 | 26477.2629 | 33037.38027 | 31446.20915 | 28047.56297 | 24835.77963 | 26317.35925 | 25325.3457 |
| POS | SM(d14:0/24:1) | 759.629246 | 480.5015 | 2774.596663 | 6079.921642 | 10000.33433 | 1177.807135 | 23882.40915 | 2470.438445 | 1330.854 | 1413.633311 | 3059.623542 | 1398.631118 | 12215.60418 | 16415.82088 | 21674.70998 | 17026.53192 | 23697.75805 | 18905.96915 | 16660.15018 | 13860.65552 | 16465.21667 | 18489.24193 |
| POS | SM(d15:0/24:1) | 773.6465167 | 496.5245 | 378.0858237 | 323.3881432 | 443.419954 | 406.3021835 | 863.8952405 | 366.8805503 | 337.4394897 | 289.0787273 | 262.6751427 | 336.8240928 | 1450.5631 | 211.1133367 | 1361.875249 | 1189.903563 | 1890.039427 | 882.4254715 | 1488.964086 | 906.660738 | 882.140852 | 895.7604207 |
| POS | SM(d15:0/24:0) | 775.6599641 | 436.0565 | 1407.206085 | 999.735483 | 1287.459105 | 686.5103678 | 2273.633242 | 1655.390141 | 654.6683995 | 500.0293492 | 861.8864783 | 627.9330262 | 5284.975443 | 6234.641658 | 7021.342608 | 5651.554435 | 7693.68323 | 6409.598038 | 5941.810135 | 5529.577723 | 5642.573855 | 5785.75482 |
| POS | SM(d14:1/26:1) | 785.6592923 | 429.927 | 523.1765395 | 218.3169057 | 345.2373425 | 174.4023792 | 934.9276063 | 380.3855982 | 133.2501963 | 163.7265086 | 251.9782365 | 202.3285495 | 3088.425952 | 2983.432573 | 4196.14139 | 3869.904017 | 5632.99671 | 4504.885837 | 6067.477362 | 4032.05455 | 2492.901948 | 2871.084193 |
| POS | SM(d14:0/26:1) | 787.6611769 | 502.373 | 23464.8848 | 9886.269478 | 20722.05652 | 11745.6608 | 63620.23088 | 27075.22078 | 4493.612185 | 4198.488743 | 7061.163327 | 7754.406365 | 7513.520098 | 10901.73831 | 15774.98228 | 10608.4205 | 14275.47285 | 9843.498905 | 8822.4262 | 7439.890345 | 8034.21139 | 8687.711372 |
| POS | SM(d15:0/26:1) | 801.6777254 | 525.31 | 485.4921803 | 266.5518275 | 528.5860132 | 207.8612965 | 834.3984107 | 525.672513 | 320.1513378 | 335.4798498 | 469.9522335 | 232.1292 | 2952.506258 | 2710.153518 | 3191.149368 | 2734.636467 | 4074.124815 | 2481.595332 | 2865.314052 | 2058.03311 | 2076.739173 | 1867.235327 |
| POS | SM(d15:0/26:0) | 803.6916437 | 469.2205 | 1036.567314 | 1212.060447 | 1218.548403 | 881.68587 | 2287.621763 | 1512.710258 | 530.984935 | 522.954425 | 792.7224345 | 651.6907172 | 2245.844785 | 2258.879702 | 3958.450313 | 3007.067175 | 4349.374158 | 3402.081918 | 2196.931707 | 2755.061253 | 2582.001258 | 3056.32739 |
| POS | SM(d16:0/26:1) | 815.6968565 | 508.783 | 1172.882353 | 667.1089118 | 952.3402663 | 555.9830353 | 1479.807799 | 1119.562017 | 589.3334217 | 528.863888 | 675.9137285 | 572.5325357 | 2933.548935 | 2553.874043 | 2638.079325 | 2248.444917 | 2841.279943 | 2153.609658 | 2314.439383 | 3058.676762 | 1949.92944 | 1992.36046 |
| POS | HexCer(d18:1/16:0) | 700.5683246 | 303.9685 | 235.8666875 | 195.8969868 | 189.529136 | 130.0128453 | 304.8978095 | 200.9843813 | 138.3864163 | 165.9039823 | 294.5522502 | 192.3566345 | 2374.251623 | 3010.428358 | 2947.375808 | 2351.454198 | 3343.705012 | 2817.5376 | 2571.548897 | 2115.851005 | 2426.644638 | 2318.155518 |
| POS | HexCer(d18:1/22:0) | 784.6627482 | 430.264 | 153.5212276 | 77.28272662 | 51.03737903 | 43.8198944 | 129.1235873 | 129.515972 | 69.3504268 | 67.57353712 | 84.83020212 | 68.19705135 | 4979.239358 | 5243.700742 | 6060.596468 | 4424.966702 | 6077.25433 | 4703.733653 | 4580.29271 | 4246.619497 | 5137.661607 | 3973.777532 |
| POS | HexCer(d17:1/24:0) | 798.6777178 | 447.488 | 420.067717 | 408.0360985 | 283.683919 | 218.1676615 | 1506.750297 | 895.1180048 | 135.8551791 | 282.3986702 | 503.8858417 | 190.9206815 | 2199.623463 | 3558.311033 | 4067.090538 | 2282.729758 | 2806.376735 | 3797.543727 | 2614.697698 | 3178.745257 | 3228.119963 | 3160.313953 |
| POS | HexCer(d18:1/24:0) | 812.6945973 | 464.7275 | 412.5593428 | 261.2383837 | 216.6637507 | 191.8253068 | 563.5339087 | 375.6837877 | 179.5399063 | 109.4738866 | 151.989364 | 208.2230058 | 2534.978343 | 2114.887942 | 2452.46992 | 1961.170737 | 3339.125752 | 2557.582727 | 2142.288635 | 1795.698505 | 2225.384048 | 2215.107163 |
| POS | PE(15:0/0:0) | 438.2605263 | 73.3905 | 26.73196528 | 26.32196215 | 23.22216658 | 26.32362608 | 21.58908122 | 23.0679395 | 22.51377808 | 25.9714561 | 22.0813405 | 24.69269948 | 29.44733877 | 27.94133945 | 35.21700158 | 31.666211 | 29.48518637 | 30.78206758 | 28.1438004 | 32.29604618 | 31.06674442 | 33.94808252 |
| POS | PE(16:0/0:0) | 452.2760522 | 82.058 | 24.80226155 | 12.87101563 | 13.59893379 | 9.790298065 | 36.47223488 | 22.84196132 | 11.39896062 | 11.95450493 | 26.12017668 | 16.55423161 | 118.3345782 | 123.1622191 | 164.3452619 | 197.862181 | 126.512682 | 124.1598919 | 125.1620691 | 136.7614666 | 137.8286685 | 151.2394542 |
| POS | PE(18:3/0:0) | 474.2603989 | 61.853 | 6.662207142 | 8.485274448 | 10.3784128 | 6.456379427 | 5.34480432 | 3.045185542 | 7.072941403 | 4.560949257 | 6.25287662 | 5.63808599 | 20.24946218 | 14.69049924 | 14.92406867 | 13.38780186 | 9.79192609 | 15.03363615 | 9.356525498 | 10.7546505 | 12.06900121 | 17.21955025 |
| POS | PE(18:2/0:0) | 476.2762076 | 70.1635 | 127.6583176 | 61.2987035 | 38.79584528 | 44.59418907 | 321.5336007 | 200.9018302 | 46.14552927 | 48.96962212 | 137.9911112 | 51.46642762 | 81.3958979 | 76.05225903 | 115.2607952 | 91.74419875 | 84.91426883 | 86.8076708 | 85.49202567 | 86.60074545 | 93.77541882 | 100.4349817 |
| POS | PE(18:1/0:0) | 478.2920201 | 84.22 | 68.1319467 | 32.3046938 | 25.36465502 | 22.68775235 | 123.4878841 | 74.38485803 | 26.30232578 | 24.7524349 | 54.47495468 | 30.51572817 | 330.4998147 | 357.666995 | 522.5370447 | 428.1052725 | 388.9312277 | 382.2430347 | 373.782741 | 395.4710145 | 385.9779663 | 444.5383978 |
| POS | PE(18:0/0:0) | 480.3073236 | 107.398 | 10.75898914 | 5.2957561 | 6.05235604 | 5.193084325 | 14.46096295 | 9.535084683 | 3.240634818 | 4.480614963 | 16.32679545 | 6.588126987 | 190.6960638 | 222.2389203 | 303.0029663 | 255.0485982 | 233.3862087 | 225.2613103 | 244.021116 | 233.9230762 | 243.2678362 | 251.8571748 |
| POS | PE(20:4/0:0) | 500.2753512 | 67.2065 | 4.073148653 | 3.202581347 | 2.852788662 | 3.064263257 | 8.666494187 | 4.690363928 | 3.725106115 | 1.937574532 | 4.256230213 | 3.033253502 | 106.5107397 | 89.91215213 | 145.6593527 | 120.9685046 | 98.69486645 | 106.8869474 | 104.1241864 | 102.1886646 | 106.5931006 | 121.947601 |
| POS | PE(P-16:0/0:0) | 436.2813031 | 93.116 | 4.858817615 | 1.884389212 | 2.190366217 | 1.818929228 | 7.072481813 | 3.249069512 | 1.425017813 | 1.913568948 | 3.005938362 | 2.354793018 | 71.89729562 | 69.98291495 | 112.2310253 | 90.55603448 | 79.37110683 | 78.42917657 | 80.23954802 | 86.5605317 | 91.11335848 | 99.05265585 |
| POS | PC(14:0/16:0) | 750.5262917 | 328.872 | 185.1044243 | 85.89061488 | 91.00716903 | 68.75682252 | 112.4655381 | 191.8020575 | 52.47556048 | 79.64594867 | 105.6422674 | 93.6185048 | 3116.975213 | 3169.70216 | 4333.508572 | 3658.815842 | 4294.671675 | 3612.911178 | 4655.242487 | 3661.18897 | 3795.504747 | 2693.552242 |
| POS | PC(16:0/14:0) | 750.5249118 | 284.126 | 8.964254255 | 2.657489382 | 3.163124913 | 2.972847218 | 12.41324734 | 5.578024993 | 2.401237893 | 4.792682388 | 4.221219143 | 3.149114123 | 66.9082043 | 89.42614867 | 86.13439458 | 83.41701878 | 86.25353643 | 84.70545892 | 78.93713503 | 82.95394322 | 90.28665245 | 86.15490503 |
| POS | PC(16:0/15:0) | 764.5397446 | 349.549 | 22.15891338 | 17.3407634 | 16.33220796 | 13.30858726 | 46.50581747 | 20.97551467 | 9.24244363 | 11.30922967 | 12.36196402 | 14.03397059 | 619.0401605 | 813.0644995 | 649.27706 | 712.9449362 | 861.8113908 | 708.7064077 | 918.7686442 | 949.6342957 | 739.4231688 | 912.2558908 |
| POS | PC(14:0/18:2) | 774.5236398 | 288.4975 | 45.41083948 | 36.92000957 | 39.29832213 | 21.48286785 | 132.3938794 | 82.64205555 | 11.62611147 | 30.83065272 | 21.61599683 | 38.40338325 | 245.2515002 | 366.4193073 | 276.3303917 | 307.6045557 | 388.8103553 | 296.7435038 | 407.4443165 | 283.9204365 | 341.6204485 | 207.424273 |
| POS | PC(14:0/18:1) | 776.5394196 | 331.2105 | 154.7714245 | 105.217737 | 103.8976272 | 65.80986715 | 310.6639635 | 150.7094578 | 68.7201134 | 57.11393813 | 93.04386398 | 112.6047881 | 1841.115358 | 2486.726917 | 2067.872652 | 2551.769625 | 2814.184623 | 2764.487067 | 2847.734105 | 2688.186485 | 2251.934763 | 1625.653133 |
| POS | PC(16:0/16:0) | 778.5564887 | 376.866 | 688.2014915 | 514.9032338 | 548.2114135 | 253.8359673 | 1138.996349 | 679.289987 | 353.8494155 | 261.3719638 | 339.6898732 | 409.7817077 | 4463.032208 | 5787.079415 | 4867.95312 | 5386.989403 | 6246.231772 | 5132.624318 | 6883.6074 | 5177.882453 | 5078.50367 | 3854.676748 |
| POS | PC(14:0/20:4) | 798.5238156 | 274.139 | 8.468794457 | 5.089957343 | 6.3531275 | 4.440324523 | 16.15903071 | 8.234117873 | 2.964507922 | 3.848612285 | 4.935409752 | 6.748319177 | 92.79198875 | 139.0602274 | 93.32205618 | 112.9054163 | 128.8658976 | 101.3477732 | 138.0652918 | 114.6057527 | 137.8033154 | 67.83310432 |
| POS | PC(16:1/18:2) | 800.5388305 | 291.226 | 27.38240188 | 15.34996709 | 16.36104894 | 12.82222217 | 64.13287872 | 21.4377727 | 7.95422246 | 8.31661974 | 11.19069857 | 16.85338088 | 149.666509 | 241.2717233 | 113.3902279 | 396.4312003 | 165.7845088 | 154.5217697 | 198.4867622 | 133.1388819 | 185.0469998 | 97.79200313 |
| POS | PC(16:0/18:2) | 802.5567752 | 342.7825 | 1308.677735 | 943.082014 | 999.4481367 | 621.162662 | 3215.680407 | 1421.443131 | 552.3829903 | 418.6595697 | 616.2254882 | 764.188986 | 1911.26655 | 6857.330827 | 5670.168682 | 2137.09391 | 7399.991515 | 2459.830447 | 2614.77054 | 2284.732653 | 2557.213215 | 4483.07794 |
| POS | PC(18:1/16:0) | 804.5611814 | 340.137 | 173.5621963 | 138.1978785 | 137.8712123 | 89.39965823 | 367.6418377 | 205.3567925 | 87.4644593 | 67.3262173 | 98.22837755 | 102.0508227 | 599.0257722 | 752.0421212 | 701.0574365 | 693.6578417 | 853.1440517 | 704.0953305 | 899.1786337 | 336.8750795 | 753.3836322 | 525.008505 |
| POS | PC(16:0/18:1) | 804.5727436 | 376.311 | 227.379758 | 186.9805582 | 192.6334765 | 72.22419702 | 870.2084505 | 226.2175718 | 63.02036763 | 49.83531522 | 90.55839958 | 133.6702901 | 11664.73135 | 15765.59199 | 13517.80839 | 14263.82654 | 16625.98404 | 13990.29061 | 18845.84728 | 13255.09082 | 13601.04013 | 10034.12623 |
| POS | PC(18:0/16:0) | 806.5769933 | 377.3845 | 79.03608625 | 78.15091618 | 54.11536858 | 30.52473413 | 125.6468169 | 58.8659404 | 40.6260366 | 19.29123015 | 29.19249135 | 52.47887852 | 1168.395825 | 1648.98498 | 1453.152386 | 1470.617395 | 1716.391372 | 1478.762358 | 1982.60915 | 1459.237784 | 1413.7808 | 1104.012223 |
| POS | PC(17:0/18:1) | 818.5857672 | 404.9255 | 16.54050656 | 14.42282151 | 14.11604253 | 9.192740613 | 33.10885742 | 21.46053818 | 8.39835455 | 8.233939588 | 15.61019715 | 12.09428224 | 687.9277698 | 906.5876403 | 806.277844 | 815.7624447 | 616.911025 | 821.5357403 | 1097.90282 | 813.148912 | 640.4715898 | 455.580923 |
| POS | PC(16:0/20:5) | 824.537685 | 286.0245 | 18.22196275 | 9.919941587 | 16.46810465 | 10.29274615 | 42.6930417 | 22.7273572 | 3.751438777 | 5.195154257 | 8.652677302 | 14.54435759 | 115.8576094 | 181.5381373 | 168.3277747 | 163.5201047 | 236.0618453 | 221.337221 | 187.0968888 | 146.4307577 | 210.772337 | 98.0004645 |
| POS | PC(18:1/18:2) | 828.5607158 | 306.502 | 29.61712612 | 11.46751736 | 13.32276099 | 15.17448822 | 36.75841507 | 18.46129688 | 7.561244158 | 12.41022062 | 18.12088182 | 10.05320233 | 41.73326432 | 65.68802552 | 51.12526025 | 47.58660792 | 56.7253273 | 50.44775137 | 54.96550185 | 49.46313208 | 50.42004583 | 41.96707553 |
| POS | PC(18:1/18:1) | 830.5842654 | 470.044 | 23.3543857 | 38.15532392 | 18.95887913 | 19.06742577 | 24.26397093 | 12.46097034 | 26.89464832 | 16.71388338 | 16.04590839 | 22.69678557 | 124.4627125 | 160.4199949 | 167.8262838 | 174.2966873 | 110.9077235 | 174.460817 | 192.3889932 | 173.1687735 | 166.8252108 | 77.31033195 |
| POS | PC(18:0/18:1) | 832.6014436 | 436.502 | 46.6670928 | 24.98550362 | 42.36146678 | 13.35761996 | 100.8788079 | 12.38390312 | 14.70128283 | 14.51685997 | 18.3310731 | 25.6344171 | 2846.456225 | 4029.916803 | 2758.238247 | 3629.6065 | 4055.071008 | 3683.867872 | 4509.096993 | 3537.153035 | 3263.235823 | 2536.335427 |
| POS | PC(20:4/18:1) | 852.5685862 | 327.182 | 16.82799125 | 7.387377988 | 6.744803423 | 4.136978298 | 15.54218533 | 9.145435492 | 4.902511082 | 4.15846876 | 4.49665144 | 6.122455068 | 776.6821347 | 1092.569292 | 1026.571684 | 986.4473753 | 1141.8849 | 962.327758 | 1322.758463 | 948.4262467 | 1011.66898 | 818.7400705 |
| POS | PC(20:3/18:1) | 854.5753374 | 326.265 | 4.516456593 | 2.39813381 | 2.723780297 | 1.858601632 | 5.505264213 | 3.201515805 | 1.996442525 | 2.130494727 | 3.358829347 | 2.077418507 | 117.5087142 | 163.5020008 | 140.7989075 | 159.2469639 | 170.6581098 | 150.7332166 | 191.0159957 | 141.5539573 | 153.3633781 | 104.2819683 |
| POS | PC(18:0/20:4) | 854.5841407 | 370.456 | 15.05660081 | 9.483239685 | 10.58961789 | 7.32062208 | 19.08190898 | 10.61869217 | 6.141005535 | 5.808147405 | 6.712083338 | 9.724225143 | 665.6904183 | 842.6142682 | 790.9341742 | 845.4675052 | 1534.744899 | 848.95414 | 1683.008315 | 850.6734733 | 849.1903882 | 598.9006743 |
| POS | PC(18:0/20:3) | 856.5991717 | 402.367 | 14.13542296 | 6.308923865 | 14.28100061 | 8.89055218 | 11.99536924 | 16.20201263 | 9.457407605 | 8.64523742 | 12.6258422 | 12.69352666 | 290.6148337 | 541.7057982 | 475.1858493 | 533.6283757 | 491.1113287 | 439.2942113 | 714.305815 | 522.4216818 | 500.1653795 | 366.9247005 |
| POS | PC(18:0/22:5) | 880.5992827 | 370.3715 | 3.627171663 | 2.821101232 | 2.903000172 | 1.918671637 | 4.514604838 | 3.144323578 | 1.828528788 | 2.150489392 | 3.328746755 | 2.608857852 | 112.6450235 | 162.7320777 | 134.7112157 | 152.844209 | 228.2198597 | 146.8647977 | 246.1617543 | 142.7550675 | 174.8184067 | 139.7446314 |
| POS | PE(16:0/12:0) | 634.4408242 | 257.325 | 10.82853299 | 1.733445068 | 2.076959357 | 2.32127875 | 11.4526012 | 4.329675662 | 1.064919918 | 2.751026402 | 2.795511138 | 3.639060785 | 75.36893285 | 97.17917443 | 87.16277552 | 95.57848362 | 109.8835946 | 94.49170033 | 115.85244 | 100.840754 | 90.78420445 | 74.96484518 |
| POS | PE(18:2/12:0) | 658.4408104 | 223.5075 | 7.969843528 | 2.60242519 | 1.850292918 | 3.900727317 | 15.58146517 | 5.515503498 | 1.38516389 | 2.213107852 | 2.714348432 | 2.59928909 | 17.10469057 | 21.252105 | 17.8377585 | 18.23105782 | 21.4913357 | 17.41415307 | 22.39470865 | 16.72790465 | 20.6763218 | 13.82214063 |
| POS | PE(12:0/18:1) | 660.4567471 | 261.721 | 20.93046693 | 5.027328648 | 5.106191253 | 8.106676107 | 27.22569378 | 10.30848293 | 2.026207365 | 5.63042242 | 6.265042838 | 7.27904008 | 109.0065423 | 150.0116778 | 128.3734318 | 145.2524613 | 166.0029068 | 137.7554301 | 163.4953547 | 135.1151809 | 140.5158354 | 106.112402 |
| POS | PE(16:0/14:0) | 662.4721363 | 305.9265 | 19.49518417 | 4.412807497 | 7.868930463 | 5.773787883 | 20.72258735 | 10.10583302 | 3.42308986 | 9.036882987 | 7.446457172 | 9.083496293 | 115.653526 | 146.7486048 | 141.7279908 | 140.1332409 | 152.7912084 | 129.1771133 | 158.2329086 | 144.7788697 | 136.8457223 | 112.9136479 |
| POS | PE(14:0/18:2) | 686.471842 | 271.2065 | 45.46482388 | 10.30262717 | 10.59284318 | 16.21867466 | 78.65138788 | 31.71782602 | 6.576169857 | 15.20184791 | 24.36057172 | 16.54912336 | 149.6594383 | 206.2504385 | 186.2763172 | 195.5020568 | 220.9687077 | 190.1973165 | 238.852661 | 197.1496645 | 198.8439212 | 149.998492 |
| POS | PE(14:0/18:1) | 688.4881889 | 309.44 | 98.21760437 | 26.45463548 | 36.39615592 | 30.93535203 | 116.020229 | 53.51493252 | 26.31884053 | 32.9753314 | 37.481698 | 49.11161405 | 502.9970523 | 634.9995807 | 573.7055072 | 607.1080007 | 660.7075347 | 587.847072 | 686.1523103 | 611.7486647 | 609.5580803 | 480.6915152 |
| POS | PE(16:0/16:0) | 690.4940062 | 309.173 | 11.38810641 | 3.540506277 | 4.920448673 | 3.459064807 | 14.39000137 | 6.082148337 | 3.771116687 | 6.64167647 | 4.705623008 | 7.465225972 | 59.8141527 | 73.89164678 | 65.0809175 | 69.35502483 | 76.23901958 | 74.03003135 | 76.86753787 | 66.31554332 | 73.24771032 | 54.41220555 |
| POS | PE(15:0/18:2) | 700.4876531 | 293.78 | 8.735381077 | 3.455546892 | 6.792887963 | 3.966358702 | 9.39346355 | 6.649739882 | 2.774044128 | 3.851585047 | 3.63290806 | 3.976771605 | 56.66195345 | 74.0928307 | 66.22944915 | 93.21093747 | 83.0461868 | 76.05669993 | 88.43494788 | 72.02080508 | 90.54823495 | 62.78068072 |
| POS | PE(16:0/17:1) | 702.5032661 | 331.9775 | 16.97342093 | 9.664963953 | 11.60638899 | 9.622115772 | 20.95424077 | 11.94374255 | 7.305099922 | 9.13603921 | 8.839126875 | 10.83457561 | 248.9198593 | 358.4018975 | 293.6511778 | 319.7597433 | 364.8989887 | 306.2063468 | 375.6898103 | 313.6117723 | 334.6963687 | 280.4629435 |
| POS | PE(16:0/17:0) | 704.518365 | 366.7955 | 5.936440685 | 4.408614227 | 10.41211204 | 6.84174091 | 10.41793421 | 5.340381828 | 3.567356152 | 6.242542042 | 3.94100955 | 4.477401603 | 79.4744538 | 96.67255885 | 95.96921608 | 101.0535074 | 109.3795905 | 98.2560823 | 113.9721192 | 104.3000026 | 96.7174063 | 78.66781522 |
| POS | PE(16:0/18:3) | 712.4880078 | 277.572 | 173.7238722 | 63.88602133 | 71.37027585 | 68.41189168 | 302.5149475 | 137.8305204 | 40.08505745 | 53.58489967 | 91.04318627 | 91.73805998 | 210.1743252 | 478.8714178 | 438.9632608 | 484.3002003 | 522.5006768 | 454.3524803 | 565.9101665 | 460.3355493 | 470.0694252 | 365.9869015 |
| POS | PE(16:0/18:2) | 714.5053257 | 318.307 | 1169.045456 | 614.979296 | 675.98023 | 403.848033 | 1977.946818 | 1043.21004 | 437.9776037 | 414.1240592 | 601.2017512 | 605.7332645 | 4364.241827 | 5367.093438 | 5397.333825 | 5603.395128 | 6132.295902 | 5408.742478 | 6593.34621 | 5596.44762 | 5533.430633 | 4408.012387 |
| POS | PE(16:0/18:1) | 716.5209217 | 353.832 | 777.1664143 | 335.2997487 | 454.8521992 | 257.7349817 | 915.3137742 | 437.7552605 | 271.7776498 | 286.6499872 | 263.419845 | 457.7728007 | 6205.909155 | 7649.730805 | 7565.70742 | 7657.742142 | 8476.54694 | 7645.770175 | 8919.58871 | 7931.341228 | 8046.883673 | 6069.797245 |
| POS | PE(18:0/16:0) | 718.5341011 | 395.334 | 31.48999107 | 27.60778013 | 22.34038992 | 16.67680728 | 41.82022293 | 24.17473207 | 11.69606012 | 16.70984728 | 15.1631837 | 21.56352502 | 231.2194537 | 277.3591858 | 266.7321985 | 292.1585385 | 304.0656265 | 261.4549827 | 331.1243327 | 288.9516048 | 279.7375343 | 215.5556628 |
| POS | PE(18:2/17:1) | 726.5031111 | 302.4355 | 14.48117516 | 9.114860375 | 9.306320968 | 9.641041593 | 35.29083365 | 12.63804402 | 6.264547668 | 8.712854478 | 7.137198017 | 9.070071643 | 74.63511568 | 93.2711129 | 80.78057122 | 83.51032125 | 119.060984 | 87.3152853 | 102.1358106 | 87.02227417 | 73.32540077 | 75.6185951 |
| POS | PE(17:0/18:2) | 728.5180869 | 339.6315 | 65.70240385 | 48.27483752 | 43.22571758 | 40.63237083 | 117.9212721 | 65.28758585 | 25.54488385 | 27.82881195 | 31.39345383 | 41.56837313 | 643.4253985 | 839.2675492 | 795.4389478 | 808.954835 | 951.9231287 | 825.206564 | 942.8698022 | 885.4903408 | 831.5063082 | 637.2180073 |
| POS | PE(17:0/18:1) | 730.5245808 | 339.19 | 8.919001263 | 6.95663461 | 4.966980282 | 5.881693347 | 26.41353515 | 7.573213592 | 4.605027587 | 5.310188013 | 9.648451205 | 6.777915825 | 93.3071144 | 116.8399628 | 109.2187231 | 112.6814268 | 126.7043734 | 112.6518226 | 124.7215796 | 108.6694257 | 116.3897098 | 84.19133792 |
| POS | PE(18:0/17:0) | 732.5407215 | 375.945 | 4.233580848 | 3.000185157 | 3.38975269 | 2.731909378 | 6.479436668 | 3.25952176 | 2.307910448 | 2.751308027 | 2.456890125 | 3.198174952 | 109.9793817 | 111.9337825 | 106.082111 | 118.7251183 | 141.4647158 | 109.7766934 | 128.1979208 | 119.477635 | 113.7366009 | 97.8980349 |
| POS | PE(18:3/18:2) | 736.4874889 | 252.129 | 32.44111382 | 11.2886617 | 21.21885882 | 11.53664442 | 58.73455552 | 30.70334913 | 7.163344577 | 10.53023075 | 13.82969306 | 19.6952167 | 47.13074237 | 34.44063563 | 54.68416268 | 53.87982063 | 69.38076903 | 37.61792187 | 73.48451073 | 60.02616418 | 62.54700623 | 47.06673413 |
| POS | PE(16:0/20:5) | 736.486935 | 276.33 | 4.73522796 | 1.946812028 | 2.745644773 | 1.874453862 | 7.604071202 | 3.622375825 | 1.243577407 | 2.005292082 | 2.679778898 | 2.937311852 | 152.3484693 | 202.6898262 | 192.9142475 | 208.9427237 | 218.1689598 | 267.7717962 | 234.8700153 | 258.38247 | 201.7137473 | 155.9399809 |
| POS | PE(18:2/18:2) | 738.5043828 | 286.9775 | 368.9061338 | 162.4375922 | 228.463956 | 129.236808 | 769.2756223 | 373.7608427 | 105.5157426 | 102.2754021 | 156.6774639 | 179.4889235 | 1013.891419 | 1310.846677 | 1183.006776 | 1375.507807 | 1515.375634 | 1332.393101 | 1646.719114 | 1287.472445 | 1289.912546 | 1028.326969 |
| POS | PE(18:1/18:2) | 740.5214859 | 322.8975 | 3757.278437 | 2895.626752 | 2130.729182 | 2169.719662 | 6468.000083 | 3970.286697 | 1328.694178 | 1299.800705 | 1899.774732 | 2157.055753 | 12057.45584 | 15407.90665 | 15123.60594 | 15939.14512 | 17232.62953 | 15428.99223 | 18500.48455 | 15572.50907 | 16067.44012 | 12213.12209 |
| POS | PE(18:0/18:2) | 742.5260922 | 322.065 | 371.6804168 | 286.1947872 | 209.0012977 | 212.4003045 | 625.6817997 | 373.7610165 | 132.8188582 | 129.5960444 | 210.0385247 | 209.7126102 | 1121.213563 | 1485.840524 | 1534.698902 | 1506.897352 | 1654.323813 | 1429.688737 | 1775.042862 | 1460.746903 | 1618.600134 | 1184.007107 |
| POS | PE(18:1/18:1) | 742.5368018 | 359.702 | 2260.673317 | 1525.585584 | 1359.400673 | 853.3421952 | 3470.388857 | 1964.04523 | 770.8583678 | 722.3974582 | 1018.39183 | 1316.997313 | 22114.10885 | 26720.8104 | 26965.85527 | 26917.03305 | 31274.0402 | 27893.87293 | 31937.61427 | 29056.52223 | 28182.8758 | 22210.66532 |
| POS | PE(18:0/18:1) | 744.5511375 | 398.6425 | 218.9239742 | 106.4914154 | 113.7784918 | 62.57938108 | 235.876741 | 133.4922225 | 68.37668442 | 92.9820539 | 88.5465289 | 116.4223263 | 7147.754783 | 9282.274018 | 8497.053643 | 9217.740625 | 10176.20155 | 9255.068543 | 10815.10497 | 9346.400492 | 8320.363722 | 6780.21315 |
| POS | PE(18:0/18:0) | 746.5565988 | 398.577 | 26.14963323 | 13.84608718 | 13.33858346 | 9.113228258 | 28.62739787 | 15.95379097 | 8.754926145 | 9.578867325 | 11.73322301 | 14.08572108 | 680.6882283 | 887.7790505 | 834.6787187 | 904.9277708 | 1002.089615 | 874.8270685 | 1061.580661 | 906.8146545 | 833.8538525 | 652.1814657 |
| POS | PE(18:1/20:4) | 764.5197612 | 312.57 | 48.72385587 | 50.79762728 | 31.18134068 | 27.88747213 | 76.3730863 | 41.17721298 | 24.53615332 | 18.26616203 | 25.18854612 | 29.40997213 | 2869.464577 | 3625.455697 | 3327.764933 | 3750.2425 | 3981.733428 | 3585.238565 | 4255.12437 | 3737.445332 | 3598.410642 | 2930.463485 |
| POS | PE(18:0/20:4) | 766.5258608 | 311.951 | 12.95835856 | 11.04097023 | 7.51687293 | 6.577579175 | 17.28032672 | 11.59240954 | 4.973709453 | 7.676997108 | 5.980764898 | 7.317004932 | 343.2299993 | 447.7123433 | 428.3201062 | 468.7613847 | 484.7289225 | 429.6671665 | 519.5296447 | 460.6257468 | 451.4982977 | 353.5985485 |
| POS | PE(18:1/20:3) | 766.5340559 | 332.36 | 61.50823655 | 42.32312632 | 46.21273938 | 26.86075195 | 105.1782177 | 55.23609847 | 23.97320302 | 24.30731962 | 30.16532975 | 35.22582143 | 1394.939732 | 1759.636928 | 1630.616625 | 1746.032645 | 1892.151008 | 1720.162498 | 2074.078523 | 1758.901547 | 1698.820672 | 1344.257915 |
| POS | PE(18:0/20:3) | 768.5408136 | 333.164 | 14.85987219 | 12.85651851 | 11.30684224 | 10.59372098 | 20.54382803 | 13.17123495 | 11.16560115 | 12.11388907 | 11.7723247 | 11.50531341 | 178.6201703 | 230.7762145 | 216.9619442 | 210.7165507 | 260.0300878 | 218.2983793 | 269.5894468 | 233.0301092 | 234.6459428 | 177.650163 |
| POS | PE(20:0/18:1) | 772.5801329 | 437.5365 | 24.58152383 | 13.34413671 | 18.48161715 | 8.174667725 | 35.32998442 | 18.11651547 | 9.385981983 | 9.664386437 | 11.83852581 | 11.59930862 | 149.8782434 | 194.212683 | 182.6966925 | 202.1430538 | 217.2173387 | 191.931976 | 231.4688118 | 194.8322942 | 169.5956058 | 140.5071804 |
| POS | PE(18:0/20:1) | 772.5881274 | 472.3405 | 15.36721209 | 14.26645941 | 13.30081241 | 1.678103738 | 27.13871158 | 19.43364095 | 2.30716601 | 8.447517587 | 14.03130401 | 7.944930423 | 36.1390632 | 57.88507318 | 42.39134527 | 41.07704703 | 41.14197777 | 41.36283633 | 43.78873173 | 37.51873843 | 40.08603242 | 35.6653556 |
| POS | PE(18:1/22:5) | 790.5353098 | 314.3185 | 21.8987615 | 10.17620489 | 7.816647742 | 8.884542268 | 25.57445143 | 13.33741246 | 5.15766215 | 5.503904437 | 10.96234436 | 7.446112165 | 368.1247447 | 504.6559293 | 445.5190927 | 481.7180693 | 553.9878247 | 469.7951703 | 581.543533 | 455.8625613 | 530.7438988 | 344.8866872 |
| POS | PE(18:0/22:5) | 792.5494476 | 351.829 | 48.83654258 | 35.79901272 | 34.05261827 | 22.74366883 | 81.3135234 | 45.80294418 | 22.79221422 | 21.66963023 | 26.79333395 | 29.77527377 | 849.8393083 | 1070.729895 | 1056.242179 | 1081.946876 | 1223.323976 | 1121.943637 | 1309.194434 | 1075.283512 | 1118.569228 | 824.5653547 |
| POS | PE(22:1/18:2) | 796.5814952 | 405.6165 | 31.20677018 | 13.09798675 | 17.62786687 | 22.8430064 | 49.16851538 | 19.44536747 | 8.682116083 | 20.92053843 | 10.88697121 | 15.41086099 | 28.41688332 | 40.20758958 | 104.2085216 | 35.26663933 | 46.66734107 | 37.87665997 | 41.53986453 | 49.22552567 | 20.06313447 | 38.06129425 |
| POS | PE(22:0/18:2) | 798.5972575 | 442.936 | 29.78697553 | 15.20725542 | 16.40739339 | 9.663958545 | 52.7913397 | 23.60024355 | 4.10964588 | 10.22451506 | 12.4025512 | 10.23233866 | 45.88027798 | 57.38102718 | 65.17136425 | 68.64419473 | 69.64430903 | 63.22832157 | 65.76615818 | 67.81545487 | 51.58784722 | 49.42506225 |
| POS | HexCer(d18:1/20:0) | 800.611509 | 473.606 | 11.48874274 | 9.191618938 | 10.44996551 | 2.003871057 | 18.15351072 | 11.68572782 | 1.928713443 | 5.177416225 | 7.73895027 | 6.341007327 | 44.4983893 | 57.84196572 | 52.23188257 | 59.38371208 | 56.04411042 | 51.09339833 | 60.45773098 | 50.76221855 | 52.39299917 | 47.16553825 |
| POS | HexCer(d19:1/22:0) | 842.6665915 | 441.273 | 4.781759178 | 6.001688375 | 2.82096707 | 2.31383782 | 7.069094053 | 2.452104652 | 7.574237468 | 3.600247327 | 4.550087317 | 4.896946355 | 872.5773408 | 1128.525909 | 945.5348575 | 1105.92714 | 1151.84066 | 954.7919313 | 1180.167995 | 1089.926014 | 932.5690067 | 724.8044538 |
| POS | PG(16:1/16:0) | 719.5023556 | 415.1975 | 4.588701828 | 3.033166912 | 3.230461803 | 3.45814291 | 4.395321367 | 3.982473213 | 3.899216473 | 3.202550725 | 2.626599245 | 2.961039897 | 122.1182415 | 125.6205704 | 183.26855 | 145.4220266 | 169.9172187 | 149.3474268 | 183.1549863 | 174.9094907 | 165.4959926 | 124.9577968 |
| POS | PG(16:1/18:1) | 745.5184288 | 418.637 | 4.018130683 | 2.534596297 | 1.69761265 | 3.202847012 | 1.774500165 | 3.017625608 | 4.134112732 | 1.92262747 | 1.735119388 | 2.17115323 | 160.4753374 | 140.2198461 | 253.5995615 | 194.236353 | 232.9365695 | 195.994456 | 227.3293218 | 200.1502365 | 222.221917 | 167.4516157 |
| POS | PG(16:0/18:1) | 747.5126625 | 261.6105 | 10.80927454 | 10.25684118 | 6.505696872 | 9.695673847 | 17.44778447 | 11.35633213 | 3.798563422 | 3.621338935 | 4.973862963 | 6.46163478 | 220.6220143 | 307.3173213 | 257.6004073 | 278.1531155 | 331.2168775 | 281.8498372 | 418.4515813 | 255.5445555 | 322.4713567 | 153.8276656 |
| POS | PG(18:2/18:1) | 771.5328156 | 421.223 | 3.380882613 | 2.22156249 | 1.859575003 | 1.409099592 | 3.440837863 | 3.04266938 | 1.465953677 | 1.564884668 | 2.145425155 | 1.777337623 | 85.14900983 | 80.74979428 | 128.4537503 | 105.3958693 | 121.3318 | 112.396744 | 123.7330375 | 103.2239079 | 113.4940318 | 74.22424892 |
| POS | PG(18:1/18:1) | 773.5271732 | 238.831 | 6.709105803 | 4.757230552 | 3.668426268 | 3.4944702 | 13.55255061 | 5.753439555 | 3.338440785 | 3.464955712 | 3.181715557 | 4.948999002 | 113.5873367 | 172.567055 | 131.8903914 | 148.1425018 | 171.7455778 | 144.9759769 | 211.9898612 | 145.8993106 | 165.5964199 | 94.34294042 |
| POS | PG(20:1/16:0) | 775.5646262 | 470.612 | 11.38776074 | 7.479293418 | 7.247403985 | 3.642782365 | 4.273422425 | 8.971123133 | 7.350073952 | 6.914872332 | 7.794581413 | 8.380375472 | 14.00153502 | 39.96253098 | 45.11774392 | 40.88241795 | 51.07746543 | 44.61503088 | 51.32483257 | 45.8364268 | 42.86180333 | 20.66839443 |
| POS | PS(18:0/18:2) | 786.521979 | 351.375 | 222.2851992 | 168.2554637 | 72.99370798 | 119.2020993 | 277.0070725 | 180.4853675 | 138.6620559 | 119.2135308 | 119.4467054 | 133.6478312 | 523.9632195 | 604.2654628 | 679.4140275 | 505.7579115 | 831.902436 | 686.0256143 | 793.3215262 | 560.3582435 | 692.6078427 | 420.7024218 |
| POS | PS(18:0/18:1) | 788.5397968 | 325.459 | 348.1974203 | 259.1789708 | 182.3329867 | 142.8024621 | 322.16688 | 261.5814733 | 115.4257679 | 156.6014476 | 160.3192868 | 160.1045517 | 6150.153868 | 8748.466803 | 6689.31751 | 6990.06175 | 10593.15464 | 7287.736953 | 9401.053522 | 6360.73079 | 9713.805098 | 3491.275623 |
| POS | PS(18:0/22:6) | 834.5153798 | 212.5145 | 48.96996362 | 27.51440947 | 57.5399482 | 19.693562 | 148.1883243 | 71.30723722 | 32.07591543 | 28.66406888 | 41.35897215 | 46.11128008 | 240.6474147 | 416.6158168 | 248.746449 | 382.4418043 | 534.6441878 | 367.4161197 | 753.518016 | 267.247652 | 474.5543123 | 171.8977012 |
| POS | PS(18:0/22:5) | 836.5315703 | 247.165 | 58.9181689 | 48.40435118 | 68.78631202 | 30.63019682 | 126.1814254 | 81.96001198 | 36.88283712 | 32.14311407 | 46.04316187 | 40.51948413 | 961.3618157 | 1505.743025 | 898.9589898 | 1149.142311 | 1916.479423 | 1270.530875 | 2385.32766 | 998.9465202 | 1453.469344 | 604.0956552 |
| POS | PS(18:0/22:4) | 838.554283 | 314.346 | 33.96386322 | 15.96754929 | 18.22248507 | 12.9648519 | 29.26256837 | 23.62774027 | 14.86904342 | 13.67633669 | 17.63911987 | 15.07156343 | 861.0921268 | 1152.468548 | 1042.988702 | 1064.533506 | 1417.023142 | 1053.174905 | 1392.252251 | 921.1306047 | 1381.060698 | 721.2231762 |
| POS | PE(P-16:0/16:0) | 674.5083123 | 377.58 | 46.62375122 | 4.810224518 | 19.81531622 | 7.907002385 | 30.15579255 | 11.95544369 | 8.703622178 | 16.06492768 | 9.074099228 | 20.35499215 | 51.88631675 | 80.83456462 | 55.63121715 | 86.4291617 | 74.29786107 | 58.09245722 | 79.60233638 | 92.30195187 | 60.03256188 | 50.46139482 |
| POS | PE(P-16:0/18:2) | 698.5087708 | 344.3285 | 657.1190118 | 118.2555431 | 196.1888332 | 77.80665343 | 715.6217733 | 197.037504 | 112.3289311 | 158.3121632 | 169.0078918 | 248.626699 | 1186.127516 | 1565.5163 | 1398.015385 | 1519.098077 | 1703.266267 | 1475.007115 | 1819.391597 | 1550.33706 | 1513.253695 | 1173.748035 |
| POS | PE(P-16:0/18:1) | 700.5245512 | 380.272 | 333.4792508 | 73.70058555 | 125.0017512 | 50.9600368 | 366.9410552 | 116.9526103 | 74.88911165 | 106.2716309 | 101.884135 | 143.4079109 | 1251.553016 | 1685.727975 | 1493.258815 | 1729.665207 | 1898.81188 | 1595.845353 | 2052.502382 | 1654.064493 | 1546.642534 | 1296.569164 |
| POS | PE(P-16:0/20:4) | 722.5090467 | 332.339 | 76.69410297 | 30.22402437 | 33.32065612 | 15.03421332 | 111.8832506 | 77.10639278 | 25.31129615 | 24.06715335 | 25.70764798 | 42.8743774 | 3335.45576 | 4304.208427 | 3848.164815 | 4258.761145 | 4730.327037 | 4079.87446 | 4971.961897 | 4238.093367 | 4218.125833 | 3318.690872 |
| POS | PE(P-18:0/18:2) | 726.5397379 | 388.5815 | 300.0190147 | 169.4999522 | 138.2628536 | 106.876147 | 438.4115135 | 181.4673173 | 75.71443602 | 118.9443193 | 149.0820402 | 177.3990453 | 1277.888316 | 1695.061858 | 1518.117364 | 1749.115113 | 1892.376772 | 1648.906962 | 3039.218778 | 2471.966502 | 1527.464459 | 1253.723263 |
| POS | PE(P-18:0/18:1) | 728.5546127 | 422.9525 | 37.88497918 | 39.43920653 | 25.03104317 | 27.3028753 | 56.6164452 | 30.36615105 | 18.20229227 | 20.83847892 | 19.89482057 | 32.06333377 | 699.0751902 | 925.836638 | 806.0770315 | 2334.713775 | 1009.793718 | 845.0604427 | 1047.199541 | 876.4768272 | 841.5490282 | 685.948093 |
| POS | PE(P-18:0/20:4) | 750.5389298 | 377.4225 | 37.86868243 | 28.31360807 | 21.34666685 | 17.02988845 | 52.19770482 | 46.97400682 | 13.54679401 | 16.17347708 | 19.4057309 | 33.09273512 | 1870.76658 | 2508.977053 | 2314.973183 | 2548.305153 | 2778.018768 | 2389.623507 | 3106.984788 | 2505.561987 | 2397.268157 | 1920.640663 |
| POS | PE(P-18:0/22:5) | 776.555019 | 378.446 | 58.62535922 | 32.30491683 | 29.22250278 | 14.50622825 | 57.57780605 | 25.42345985 | 34.28344452 | 28.40455925 | 32.42191932 | 46.2123102 | 216.1471338 | 327.6564878 | 265.8570498 | 300.4967903 | 332.9860495 | 308.6283785 | 359.7328828 | 287.2888202 | 283.4689472 | 229.2725555 |
| POS | Hex2Cer(d14:0/18:1) | 878.5837819 | 328.056 | 5.556759225 | 3.66338916 | 4.226658738 | 3.600103657 | 7.388847027 | 4.696647168 | 3.348186212 | 3.068240223 | 4.71728197 | 3.611898722 | 197.7360985 | 270.3275277 | 204.3105055 | 233.7933613 | 272.7430228 | 405.8008163 | 296.0970253 | 256.710884 | 252.0685018 | 201.4985363 |
| POS | Hex2Cer(d14:0/18:0) | 880.5926129 | 244.9635 | 27.61870408 | 41.94096783 | 34.00652367 | 52.45882425 | 80.11828437 | 54.11283853 | 8.879498705 | 17.08403772 | 40.67530458 | 19.39908802 | 31.1931702 | 40.08865728 | 32.78035805 | 38.06207162 | 45.36964885 | 32.93710272 | 45.82845975 | 36.27115978 | 42.15661853 | 29.07284677 |
| POS | Hex2Cer(d14:1/20:0) | 906.6098996 | 274.471 | 49.25703383 | 46.00102517 | 33.49955427 | 36.93332007 | 87.61644207 | 53.7468488 | 13.69530177 | 23.79300572 | 54.8812911 | 23.15810523 | 3748.202768 | 5541.888785 | 4291.298233 | 5172.643433 | 5790.860752 | 4800.067418 | 6410.338802 | 5002.396915 | 5002.528973 | 3646.273297 |
| POS | Hex2Cer(d14:0/20:0) | 908.6244457 | 291.282 | 32.95724605 | 46.1673305 | 30.05284428 | 71.67183818 | 62.07422038 | 69.1739851 | 14.65663841 | 19.2489725 | 56.08564483 | 19.68050292 | 326.6519222 | 463.3810655 | 412.5506828 | 467.8397072 | 492.2662855 | 432.705607 | 560.0754745 | 436.7295628 | 449.6931685 | 306.9036992 |
| POS | Hex2Cer(d14:1/22:0) | 934.6386924 | 320.356 | 10.24069241 | 16.34505599 | 12.37585163 | 10.88952759 | 18.86233445 | 19.37349152 | 5.627622222 | 6.924849188 | 13.41534628 | 8.273332437 | 245.3731972 | 288.6985305 | 282.4958888 | 306.9805137 | 334.9680992 | 276.5873632 | 351.3960662 | 299.3257737 | 317.2811882 | 215.687969 |
| POS | Hex2Cer(d14:0/22:0) | 936.6534822 | 336.472 | 9.398692548 | 25.89125538 | 17.95486373 | 29.92249148 | 22.08612343 | 33.4157196 | 7.547070327 | 11.05372505 | 18.99393405 | 8.940094867 | 55.82250832 | 62.51941218 | 64.68621247 | 68.98929245 | 70.05675832 | 60.87881877 | 78.14118857 | 67.0316708 | 67.16305447 | 48.01788842 |
| POS | Hex2Cer(d14:0/24:1) | 962.6695824 | 364.015 | 11.05634754 | 24.3905088 | 13.75833524 | 10.31380952 | 27.0607442 | 17.09102068 | 6.514237735 | 6.206260907 | 14.05872759 | 8.001754597 | 232.220095 | 270.5277152 | 284.855041 | 292.8336127 | 304.2999407 | 274.3591315 | 337.2874288 | 304.4273172 | 305.0041765 | 213.24893 |
| POS | Hex2Cer(d14:0/24:0) | 964.6847297 | 377.032 | 19.08043828 | 56.59694312 | 37.24926035 | 56.61958057 | 54.08106075 | 66.46686848 | 17.0125449 | 11.46919823 | 31.181885 | 15.33790596 | 69.5545529 | 72.31675335 | 74.20931932 | 77.9627367 | 91.86486887 | 69.36478887 | 101.6922756 | 78.83838678 | 74.85787563 | 58.99414163 |
| POS | Hex2Cer(d15:0/24:1) | 976.6856408 | 384.8965 | 3.221768327 | 5.731035598 | 4.027387415 | 4.039822847 | 7.664409938 | 5.551271755 | 3.138614018 | 3.478520618 | 5.044626982 | 2.570562538 | 137.1829276 | 175.712393 | 171.4015358 | 202.3169252 | 204.662252 | 161.0116006 | 221.4519685 | 182.097795 | 170.8191012 | 128.3465533 |
| POS | Hex2Cer(d14:0/26:1) | 990.7012125 | 402.9875 | 18.61949825 | 36.29730152 | 24.33951103 | 15.82696832 | 51.32613535 | 33.28296137 | 10.95859155 | 9.870875385 | 25.23195993 | 14.07939789 | 976.3342553 | 1206.595219 | 1053.276387 | 1232.524581 | 1310.694716 | 1098.42596 | 1428.843735 | 1237.682959 | 1104.191972 | 831.3942022 |
| POS | Hex2Cer(d14:0/26:0) | 992.7155456 | 416.87 | 20.26074995 | 61.1663438 | 53.22141133 | 81.87538852 | 62.9459967 | 94.6222214 | 15.83230507 | 14.92849023 | 40.88807507 | 20.10630095 | 92.33983035 | 119.8947487 | 103.4652283 | 122.6191598 | 104.6021688 | 106.5229184 | 137.8912275 | 117.419072 | 109.6678433 | 84.60869108 |
| POS | Hex2Cer(d15:0/26:1) | 1004.716191 | 420.392 | 9.64026159 | 15.60066397 | 10.9349898 | 8.740248688 | 20.61883013 | 11.40021704 | 7.48153398 | 6.944755112 | 11.03366005 | 6.498334707 | 404.179452 | 499.8526102 | 476.2608355 | 535.7389737 | 527.276158 | 462.3333468 | 591.3956938 | 514.8501288 | 478.1150672 | 386.7411233 |
| POS | Hex2Cer(d16:1/26:1) | 1016.715762 | 401.3015 | 34.56056408 | 50.54429682 | 39.55704503 | 18.32989897 | 61.86946472 | 33.99472828 | 14.58795411 | 17.31991973 | 34.00352547 | 21.72208992 | 514.9333662 | 753.9855157 | 597.5945968 | 746.4105913 | 739.7614663 | 676.6891052 | 907.5853785 | 709.6209775 | 687.8107733 | 484.6926907 |
| POS | Hex2Cer(d16:0/26:1) | 1018.732252 | 437.346 | 17.37809877 | 35.43274672 | 20.5763952 | 15.16767951 | 41.07441238 | 22.67710107 | 8.624056925 | 10.80117068 | 17.2147155 | 11.2700577 | 888.4125942 | 1118.44913 | 929.7351638 | 1161.913311 | 1023.885433 | 951.6878997 | 1339.818171 | 1131.322436 | 963.3168937 | 795.4919935 |
| POS | Hex2Cer(d17:0/26:1) | 1032.745287 | 453.525 | 1.938196378 | 4.007304953 | 2.596697205 | 10.83370642 | 7.088278285 | 3.056033617 | 1.312340192 | 0.988170107 | 2.538889113 | 1.654834225 | 62.63442767 | 72.93623565 | 60.7443213 | 75.15674825 | 60.92754865 | 68.34294407 | 74.81922792 | 65.9606494 | 63.91680953 | 53.3493381 |
| POS | PA(18:2/19:0) | 713.5258036 | 360.255 | 36.74446163 | 14.95031264 | 28.49403993 | 12.14426886 | 93.38259303 | 47.81010877 | 8.807600162 | 15.52016177 | 22.26246128 | 21.18610955 | 164.1032091 | 226.2898063 | 196.5624367 | 214.0488755 | 249.6366397 | 217.0359045 | 268.3941738 | 218.3499732 | 226.9828553 | 174.806906 |
| POS | PA(18:1/25:0) | 799.6089105 | 473.251 | 26.69343983 | 25.31997165 | 25.6050688 | 14.984447 | 45.19027068 | 33.71621302 | 7.952640928 | 15.1828465 | 22.55547028 | 13.23194147 | 38.78852148 | 40.57754788 | 40.98605888 | 37.61783862 | 39.07403107 | 39.33169028 | 42.34335687 | 41.3872109 | 34.57967068 | 37.34229302 |
| POS | PI(14:0/18:1) | 807.496123 | 203.532 | 5.344046103 | 4.329447058 | 8.373964328 | 4.187513635 | 20.42376263 | 11.13582555 | 4.2111221 | 4.912302113 | 5.663224283 | 4.98546433 | 56.76856543 | 86.44575915 | 54.19938652 | 66.75311643 | 116.0658291 | 75.50568975 | 133.580964 | 54.85294682 | 86.23618507 | 39.64606438 |
| POS | PI(16:0/16:0) | 809.5124961 | 242.219 | 4.786868573 | 4.45716393 | 5.46676626 | 3.274559842 | 10.8555581 | 7.082098768 | 2.785364832 | 3.562650492 | 4.81462081 | 3.524860307 | 75.0972529 | 117.9726534 | 108.8020561 | 91.0185558 | 146.5135582 | 93.8362006 | 352.0409408 | 76.62696922 | 117.6828122 | 49.42090387 |
| POS | PI(16:0/18:1) | 835.5275529 | 247.13 | 29.74335968 | 35.1265638 | 49.6966323 | 21.34788238 | 98.2534517 | 54.82422567 | 26.68232218 | 22.58144102 | 31.65672295 | 29.55850325 | 764.0869495 | 1249.71836 | 725.099329 | 953.0974642 | 1547.562312 | 1076.713656 | 1942.062882 | 835.1235487 | 1198.697105 | 490.1811797 |
| POS | PI(18:0/16:0) | 837.5425292 | 285.885 | 12.25302685 | 7.60925001 | 7.784343112 | 4.496554188 | 12.61979534 | 11.08068753 | 4.252914325 | 4.815027128 | 6.429778078 | 5.477594598 | 510.0088135 | 295.7392107 | 574.7264422 | 610.9323145 | 948.431733 | 240.0244988 | 411.2048857 | 559.4243238 | 295.8791562 | 284.0586693 |
| POS | PI(17:0/18:1) | 849.5430592 | 269.039 | 5.32636487 | 6.417014852 | 3.3435461 | 3.830934718 | 13.65596855 | 5.705220205 | 3.136422655 | 3.075932252 | 3.725920747 | 3.498453518 | 113.7823524 | 182.02039 | 125.5430639 | 142.6979922 | 221.4524615 | 158.2864572 | 278.5305402 | 124.4394844 | 175.858361 | 83.86290017 |
| POS | PI(16:0/20:4) | 857.5122873 | 206.088 | 5.234580137 | 2.057221193 | 3.607919698 | 1.41421006 | 8.398508848 | 2.958229433 | 1.71904731 | 1.981520848 | 2.736948105 | 2.619736073 | 352.4392972 | 595.4189378 | 338.6214283 | 431.0790425 | 721.4273753 | 455.5819727 | 931.1110877 | 370.8171273 | 506.6040377 | 212.8807648 |
| POS | PI(18:1/18:2) | 859.5276772 | 218.579 | 66.9817927 | 46.59172773 | 99.74717097 | 37.96158982 | 277.3833843 | 118.4084959 | 50.74743297 | 36.60646367 | 60.58142153 | 42.47464997 | 514.4710055 | 892.1190743 | 487.3910947 | 641.909819 | 1050.731908 | 694.5601322 | 1346.808361 | 547.4057768 | 777.9139855 | 308.5232292 |
| POS | PI(18:0/18:2) | 861.544879 | 256.0435 | 249.7478688 | 403.0203422 | 416.6713373 | 236.248648 | 1036.619203 | 455.9343822 | 169.567516 | 155.5897701 | 183.8356823 | 220.1180777 | 2183.036428 | 3629.1558 | 2235.665415 | 2746.270583 | 4503.324673 | 3182.675342 | 5745.296277 | 2384.952647 | 3543.469458 | 1423.360124 |
| POS | PI(18:0/18:1) | 863.5594336 | 290.348 | 52.96291637 | 105.7604084 | 89.72289578 | 51.65594978 | 205.8454925 | 112.0921609 | 56.07076662 | 41.08260368 | 61.89700585 | 54.58425588 | 1118.215687 | 1823.364677 | 1203.276106 | 1493.628378 | 2356.730407 | 1658.213159 | 3034.383517 | 1343.297177 | 1804.0315 | 723.6751402 |
| POS | PI(18:1/20:4) | 883.5266745 | 210.941 | 11.83038981 | 8.48862828 | 10.8544552 | 5.110279397 | 27.99087488 | 10.3954217 | 9.013789898 | 6.005801417 | 6.868244907 | 10.3797344 | 1202.625206 | 2085.316812 | 1176.261313 | 1454.68191 | 2608.13507 | 1624.941566 | 3301.73929 | 1314.156879 | 1942.683193 | 682.5256577 |
| POS | PI(18:0/20:4) | 885.5337833 | 210.762 | 3.402064177 | 2.082455437 | 3.012305002 | 1.876036385 | 7.043817 | 2.616384112 | 2.082509343 | 1.800457723 | 2.109219398 | 2.591655163 | 185.5588993 | 283.9745705 | 184.2580443 | 231.9193963 | 379.4924927 | 253.6685342 | 462.1529357 | 198.1305423 | 292.8717225 | 122.6252856 |
| POS | PI(18:1/20:3) | 885.5430135 | 229.067 | 8.264713857 | 4.545798135 | 6.926161525 | 4.434453653 | 18.73943025 | 7.556221963 | 3.74124079 | 4.738576237 | 4.402109455 | 5.326467035 | 386.8577262 | 624.380938 | 387.544411 | 491.1604347 | 795.7748647 | 552.3741643 | 1039.099112 | 424.947669 | 597.7090595 | 254.696689 |
| POS | PI(18:0/20:3) | 887.5578137 | 283.266 | 4.943878857 | 2.938680648 | 3.617891388 | 2.21113482 | 6.991458513 | 3.27865518 | 1.024541752 | 1.960466325 | 2.455679935 | 3.079027348 | 226.3096692 | 354.9865758 | 232.9016718 | 299.8978848 | 425.3209382 | 319.6396243 | 585.8554848 | 262.163777 | 360.548492 | 157.6097393 |
| POS | PI(18:0/22:4) | 913.5735814 | 280.293 | 2.658109452 | 2.92807319 | 2.719758453 | 2.019541158 | 3.655419595 | 2.400003687 | 1.311157508 | 1.631099922 | 2.102045102 | 2.001184153 | 120.9596124 | 202.1690002 | 139.0910665 | 168.0130105 | 240.7228798 | 175.3058117 | 299.7487062 | 149.8833669 | 203.894584 | 87.19935955 |
| NEG | PC(P-20:0/15:1) | 758.6155482 | 453.576 | 447.1481368 | 258.227558 | 273.9661333 | 220.2861677 | 540.2900838 | 204.1186333 | 218.2765213 | 190.2226112 | 319.9956045 | 241.1953933 | 1927.083417 | 2190.520617 | 2576.316563 | 2073.991245 | 2717.855772 | 2250.679228 | 1864.402315 | 1822.315127 | 2070.48079 | 2085.146882 |
| NEG | PC(P-20:0/17:0) | 788.6713935 | 435.639 | 569.5676587 | 382.7968957 | 481.5316488 | 243.3962578 | 851.5723077 | 631.6528288 | 254.0507635 | 209.2775427 | 348.933574 | 255.96629 | 1210.746154 | 1006.026196 | 1740.54806 | 1276.058083 | 1698.19086 | 1462.796171 | 1017.202904 | 1026.59666 | 1208.780333 | 1388.047134 |
| NEG | PC(P-20:0/18:2) | 798.6572707 | 423.8045 | 112.5730905 | 69.58823187 | 67.38697123 | 44.61051318 | 119.6938938 | 103.0034672 | 89.55921577 | 45.43600088 | 83.00439482 | 53.46943543 | 525.5134603 | 512.7350883 | 757.0523137 | 512.7756233 | 724.2111065 | 682.8251293 | 529.8054075 | 495.2548303 | 505.0418693 | 522.6664968 |
| NEG | PC(P-20:0/18:0) | 802.6879924 | 475.1745 | 670.0243927 | 1291.44357 | 1286.043041 | 959.024694 | 2501.856873 | 1578.566872 | 588.5643898 | 365.1863088 | 581.7687475 | 698.9589742 | 3209.983912 | 2667.460015 | 3390.008348 | 2711.09144 | 3757.469252 | 3636.746387 | 2613.5491 | 3147.632657 | 2471.001108 | 2785.290177 |
| NEG | PC(P-20:0/20:1) | 828.7037848 | 478.754 | 796.9168815 | 866.9939027 | 1279.185389 | 785.7985995 | 2743.336465 | 1213.723504 | 149.8333172 | 249.6679673 | 682.8543128 | 595.3522473 | 1045.476509 | 809.4831897 | 1153.363334 | 778.75456 | 1397.443734 | 826.0989825 | 666.1478972 | 636.5654768 | 734.6586965 | 773.3523418 |
| NEG | Cer(d15:0/24:1) | 608.5929817 | 457.842 | 82.04601 | 32.38120733 | 23.76659398 | 15.93057645 | 0 | 29.40517492 | 15.42367399 | 42.70646123 | 42.64686955 | 22.83774955 | 1089.173853 | 968.5650943 | 1136.722307 | 694.4561422 | 919.2551695 | 979.4426013 | 911.8496388 | 645.9663488 | 808.7827563 | 549.2839832 |
| NEG | Cer(d16:1/24:1) | 620.5932083 | 440.323 | 172.7153285 | 29.98511632 | 55.14759708 | 23.85664903 | 69.50234473 | 59.50494568 | 35.07747348 | 35.19659818 | 39.5830957 | 39.94713235 | 1176.595613 | 1761.356727 | 1545.737915 | 1460.153573 | 2083.778088 | 1727.86515 | 1596.988669 | 1112.020614 | 1395.870177 | 1222.955022 |
| NEG | SM(d15:1/22:0) | 745.6046265 | 435.443 | 560.2910712 | 357.5009228 | 443.901072 | 259.3423138 | 711.7700752 | 522.6403947 | 250.9984635 | 191.5401768 | 317.1533928 | 256.909199 | 1727.685742 | 2130.96035 | 2743.005 | 2071.313158 | 3458.439005 | 3244.778563 | 2471.251868 | 1711.790393 | 1936.861302 | 1892.238735 |
| NEG | SM(d17:0/26:1) | 829.7070362 | 478.754 | 805.7233872 | 820.7510018 | 1277.853455 | 742.6315275 | 2569.619093 | 1126.521195 | 460.6539097 | 272.1509175 | 658.8092372 | 515.2493542 | 803.463468 | 711.2961318 | 928.151607 | 743.2796868 | 1078.175795 | 823.9133432 | 689.736809 | 641.3258347 | 631.1409483 | 799.1878243 |
| NEG | PC(24:0/4:0) | 722.4941674 | 275.5675 | 46.65268722 | 10.72445252 | 9.613144507 | 15.58258702 | 91.6760689 | 27.66740693 | 7.081164378 | 42.20917158 | 18.1775238 | 14.8893376 | 773.2439303 | 966.7229025 | 869.6676135 | 862.4467282 | 1057.48871 | 863.8536097 | 1085.937381 | 985.9033255 | 889.852044 | 647.417509 |
| NEG | PC(12:0/18:1) | 748.507794 | 281.829 | 25.13497317 | 7.186640495 | 7.569247252 | 7.285443367 | 43.90768113 | 23.20524097 | 4.718761455 | 6.586287193 | 8.354677562 | 9.780254037 | 283.931461 | 372.878845 | 232.9997033 | 335.4336433 | 304.8578153 | 250.0337458 | 452.4928837 | 245.5496655 | 364.3558768 | 187.6972365 |
| NEG | PC(18:1/12:0) | 748.5157301 | 261.226 | 3.83290585 | 2.313575648 | 2.399327218 | 1.993295315 | 6.701652795 | 4.03081713 | 1.523299753 | 1.758129907 | 1.97146762 | 2.289288107 | 74.33192188 | 101.0071662 | 84.91482048 | 90.76161992 | 112.7479416 | 93.74244948 | 133.732838 | 85.53832462 | 99.12643887 | 57.80789975 |
| NEG | PE(14:0/20:4) | 710.4722218 | 261.5095 | 2.776362108 | 1.519484663 | 1.47802574 | 1.365595893 | 5.615612457 | 2.311408355 | 0.968777335 | 1.659816225 | 1.92201649 | 1.673657365 | 68.14568617 | 94.85222647 | 81.64417373 | 95.3791641 | 111.3458248 | 92.61738178 | 110.3917459 | 93.3615756 | 91.71477418 | 70.502563 |
| NEG | PE(17:0/20:3) | 754.5331212 | 346.6705 | 12.29114204 | 13.91967276 | 13.19151499 | 13.28055017 | 21.19895625 | 20.40693643 | 8.528462688 | 8.96064535 | 7.545105828 | 8.844159147 | 110.2324217 | 124.7773194 | 112.6847313 | 119.8197859 | 133.4323285 | 118.2538578 | 139.565814 | 122.453358 | 121.1459248 | 163.3693806 |
| NEG | PE(19:0/18:2) | 756.5492389 | 381.35 | 25.72384842 | 15.64219963 | 18.25397687 | 11.9294744 | 36.73073883 | 20.42946218 | 10.20004079 | 11.67854358 | 15.93285925 | 16.31996806 | 153.566164 | 196.1948178 | 192.6142207 | 200.0810445 | 228.98439 | 190.5290322 | 246.4296592 | 194.3576122 | 195.0026348 | 153.8120865 |
| NEG | PE(19:0/18:1) | 758.5650663 | 417.751 | 6.276098437 | 2.935888097 | 3.830052273 | 2.309826123 | 6.731988837 | 4.22366772 | 2.082643098 | 2.973581798 | 3.444515702 | 3.961429028 | 85.52130723 | 117.0088787 | 103.6391911 | 121.5438643 | 142.7812839 | 98.65783217 | 137.6459878 | 101.3179908 | 100.5432154 | 91.14798477 |
| NEG | PE(22:6/16:1) | 760.5089481 | 280.829 | 49.18278972 | 9.619259937 | 15.17513614 | 8.457718592 | 49.75964725 | 20.56805752 | 11.52472674 | 25.06997743 | 12.38855533 | 28.2540096 | 355.9445368 | 503.18795 | 383.1605475 | 406.053729 | 677.3671942 | 453.8266012 | 585.2668713 | 364.1837192 | 588.4344837 | 203.4346998 |
| NEG | PE(18:2/20:4) | 762.5044188 | 279.129 | 10.83210773 | 9.517846875 | 11.94347181 | 5.251516515 | 17.34651627 | 8.6495098 | 5.370978388 | 4.209723083 | 5.19490248 | 5.623313598 | 482.940763 | 776.6222138 | 646.1366552 | 648.8818955 | 848.1395238 | 628.3439817 | 894.8172728 | 644.613333 | 638.0870368 | 547.287985 |
| NEG | PE(18:1/20:1) | 770.5580495 | 373.3195 | 20.48301052 | 9.21979597 | 15.27013571 | 9.824521232 | 29.0064258 | 13.98341977 | 7.816985033 | 12.93596403 | 8.789793448 | 14.05772827 | 164.5730014 | 222.7468295 | 199.8357045 | 219.8499623 | 254.1933137 | 211.8790978 | 264.3296607 | 213.188328 | 263.7239803 | 168.3859887 |
| NEG | PE(22:5/18:0) | 792.570584 | 404.754 | 22.68727367 | 23.4991576 | 22.7050979 | 13.50952479 | 46.60883193 | 18.19449643 | 10.38174996 | 11.75655194 | 14.92140726 | 17.25611987 | 565.1029467 | 433.6222862 | 627.5631957 | 687.3246117 | 901.385949 | 663.4745642 | 864.1600397 | 707.9292677 | 655.8512238 | 494.3576935 |
| NEG | PE(22:4/18:0) | 794.5627944 | 385.7615 | 3.57775427 | 3.48599649 | 2.732112697 | 2.714635152 | 4.472669935 | 3.074094118 | 2.639315092 | 2.923065367 | 2.956654738 | 3.61553658 | 342.0356143 | 445.9428273 | 439.5522542 | 472.7160492 | 520.0016627 | 429.9791093 | 554.6662028 | 449.8863673 | 415.2603235 | 352.5455498 |
| NEG | PE(24:0/16:0) | 802.6344681 | 402.2895 | 3.654625947 | 4.309692855 | 3.206197957 | 2.393362013 | 6.665718518 | 4.944039622 | 5.509547973 | 2.592664898 | 3.884957055 | 4.06417522 | 40.01799173 | 51.13540987 | 83.75381738 | 49.32521982 | 84.63192018 | 47.70245385 | 60.91030902 | 56.19924458 | 48.84330132 | 40.49288588 |
| NEG | PE(22:6/20:3) | 812.5360816 | 398.795 | 24.46095647 | 11.97303551 | 13.48197294 | 8.183767822 | 22.48059773 | 14.71129964 | 8.078252427 | 8.70113727 | 11.34282383 | 13.06024127 | 286.6721137 | 285.1608688 | 342.0121847 | 301.441214 | 344.7444507 | 333.8281355 | 350.8728677 | 310.322786 | 327.5679692 | 238.4980918 |
| NEG | PE(24:0/18:0) | 830.6522923 | 425.4275 | 61.33752717 | 56.34320875 | 17.10522332 | 42.81615632 | 81.14582382 | 55.16792663 | 63.67179463 | 38.55336393 | 40.94915298 | 59.03898152 | 399.9963258 | 318.7279057 | 449.8061425 | 326.956467 | 322.8759038 | 321.1146822 | 399.9838962 | 446.0213203 | 313.7670038 | 245.1792695 |
| NEG | PG(20:0/16:0) | 777.5593101 | 378.7685 | 157.2068809 | 98.87710602 | 120.726521 | 62.4020953 | 317.5505907 | 141.8758705 | 49.99875502 | 50.1827807 | 72.08060955 | 124.4095125 | 179.6472175 | 248.7624352 | 333.516479 | 373.2337737 | 433.8003705 | 242.5583955 | 303.8335977 | 351.3420102 | 234.682124 | 211.3531513 |
| NEG | PS(18:1/18:2) | 784.5080814 | 253.3105 | 295.6935255 | 187.7387007 | 210.2610423 | 78.9888915 | 503.3628453 | 267.5768952 | 149.5405407 | 194.6299643 | 163.6569375 | 220.174848 | 1928.96948 | 2943.431973 | 2133.946505 | 1759.495183 | 3588.364157 | 2401.608927 | 3708.595677 | 2010.9722 | 3090.040078 | 1169.248439 |
| NEG | CL(16:0/16:0/18:1/20:0) | 1434.039861 | 354.5745 | 5.352791083 | 2.08834468 | 3.539232112 | 1.484088892 | 7.145674867 | 3.226144415 | 2.373291285 | 2.211745608 | 1.542715125 | 3.154456027 | 107.6694446 | 143.4151282 | 125.2996305 | 135.3625402 | 152.3269999 | 130.5954689 | 162.6673775 | 132.8732802 | 131.8572701 | 104.5513449 |
| NEG | CL(18:1/18:1/18:1/18:1) | 1456.02432 | 321.207 | 24.12313812 | 12.49109048 | 12.28215629 | 7.820594602 | 52.93883773 | 24.45747968 | 7.356930478 | 5.599232955 | 7.944115188 | 10.86485099 | 108.7786956 | 146.2006105 | 116.3732196 | 133.0677992 | 150.7427826 | 117.6118895 | 202.9760688 | 132.8501705 | 129.5633471 | 93.98740847 |
| NEG | CL(16:0/18:1/18:1/20:0) | 1460.057317 | 356.714 | 9.880961632 | 4.474378818 | 5.2997247 | 3.624311765 | 17.2304295 | 6.449323777 | 3.65893892 | 2.974318223 | 3.137359963 | 5.307489177 | 275.034929 | 350.750406 | 311.1333787 | 359.3750672 | 367.473388 | 353.8779967 | 386.932207 | 337.0941643 | 318.3475947 | 261.7749905 |
| NEG | CL(18:1/18:1/18:1/20:2) | 1482.039761 | 322.876 | 43.54085038 | 32.72161742 | 22.98703462 | 23.42693083 | 113.7791862 | 57.16492822 | 12.10671589 | 11.60904157 | 15.75169593 | 22.06686328 | 179.2921788 | 244.173522 | 197.8177122 | 237.6234987 | 249.8076818 | 246.4874467 | 282.6835697 | 211.6991008 | 226.4640455 | 174.9176747 |
| NEG | CL(18:0/18:0/18:0/20:4) | 1484.049348 | 354.766 | 5.807737608 | 3.106781005 | 3.279868347 | 2.245648643 | 8.98340593 | 4.194096233 | 1.982020758 | 1.658017398 | 2.398855163 | 3.09880939 | 122.3441922 | 166.8886553 | 132.1717244 | 157.7220785 | 157.851222 | 152.1596964 | 165.3074802 | 135.0475825 | 130.2621135 | 118.0410098 |
| NEG | CL(18:1/20:0/18:1/18:1) | 1486.069345 | 359.277 | 20.18658383 | 11.31616715 | 10.12751613 | 5.737722312 | 52.02846353 | 17.84195955 | 5.586005392 | 4.653920487 | 5.991852367 | 9.222041477 | 619.0801842 | 819.0458115 | 704.0783062 | 777.8267912 | 857.012169 | 758.2618078 | 946.3444207 | 744.6378255 | 732.1951932 | 618.2180067 |
| NEG | CL(18:0/18:0/18:0/20:1) | 1490.102535 | 398.3785 | 1.346563925 | 0.203412217 | 0.245444733 | 0.591438273 | 2.346132925 | 0.21902343 | 0.573566563 | 0.25847887 | 0.222397793 | 0.1959937 | 114.4006599 | 161.2678902 | 124.4408681 | 146.2835359 | 161.9092661 | 141.6271804 | 175.836347 | 136.0359657 | 247.6332643 | 109.2647982 |
| NEG | CL(18:2/18:2/20:1/20:3) | 1504.022539 | 322.065 | 22.59119063 | 16.7734699 | 12.44656749 | 13.23372369 | 58.83948123 | 27.86258263 | 6.305641315 | 6.977929013 | 7.546116773 | 11.75781554 | 87.78155242 | 93.87389462 | 98.85301507 | 130.1196963 | 146.879639 | 99.16588868 | 157.000848 | 132.065275 | 110.478259 | 76.61826188 |
| NEG | CL(18:1/18:1/20:1/20:2) | 1510.06788 | 358.259 | 5.34207168 | 3.291138238 | 3.94586423 | 2.286549008 | 11.63687069 | 5.219863432 | 1.984713408 | 1.825027882 | 1.433967872 | 3.039275545 | 190.1135415 | 206.7579478 | 149.8346181 | 219.2390892 | 209.6208433 | 205.0337628 | 239.2483093 | 208.500895 | 208.137159 | 169.4462685 |
| NEG | PE(P-16:0/16:1) | 672.4933481 | 334.887 | 102.9131733 | 17.51315073 | 49.65152347 | 14.72266831 | 175.586077 | 53.92950832 | 24.96715278 | 46.11740722 | 51.27702207 | 61.72225935 | 328.9796403 | 365.3280175 | 367.1539877 | 396.9526513 | 453.0380327 | 351.9092857 | 429.7223698 | 348.1165005 | 397.2621472 | 271.5705853 |
| NEG | PE(P-16:0/18:3) | 696.4925079 | 309.092 | 33.18733662 | 7.037168268 | 13.66502059 | 9.591282847 | 47.3079239 | 13.5934002 | 6.508755847 | 13.37764814 | 12.3514272 | 22.49050522 | 180.1448373 | 220.9114108 | 316.4384533 | 350.4695092 | 258.1639648 | 232.9665385 | 278.8963802 | 228.472823 | 224.6699653 | 183.7553327 |
| NEG | PE(P-16:0/20:5) | 720.4929239 | 299.673 | 3.544103517 | 2.165852593 | 3.285373898 | 1.996752242 | 23.6674295 | 11.76507547 | 1.808881542 | 2.667769685 | 3.842223875 | 10.23381418 | 256.7259035 | 400.551528 | 298.143891 | 373.7879418 | 445.5741137 | 385.034639 | 481.0407002 | 366.9900343 | 332.341935 | 262.8705638 |
| NEG | PE(P-16:0/20:3) | 724.5239817 | 349.992 | 390.1170718 | 142.9566347 | 177.5606042 | 90.25557623 | 429.5216133 | 174.2158343 | 69.40137998 | 139.0751164 | 155.4852928 | 180.7204393 | 892.6148307 | 1216.274129 | 1131.559333 | 1227.705656 | 1497.739365 | 1160.281589 | 1374.931427 | 1471.687094 | 1281.358899 | 914.1059073 |
| NEG | PE(P-16:0/22:5) | 748.5252784 | 335.74 | 57.85607123 | 35.85252407 | 33.59354182 | 23.3143671 | 88.34721305 | 35.43934467 | 21.19147453 | 23.60566128 | 23.67737113 | 35.59730957 | 1154.537089 | 1636.341613 | 1377.86231 | 1557.844708 | 1582.051618 | 1459.189591 | 1795.32202 | 1493.951705 | 1496.968995 | 1218.008308 |
| NEG | PE(P-18:0/20:3) | 752.5468702 | 377.558 | 15.44439034 | 6.080756537 | 3.786160962 | 3.768036433 | 14.58824075 | 6.852816023 | 2.852556225 | 3.710902608 | 5.594142812 | 10.72480804 | 277.7264073 | 411.8441912 | 282.7240015 | 433.1680255 | 479.9631668 | 422.2810412 | 481.1475467 | 295.7872187 | 282.556006 | 307.5466643 |
| NEG | Hex2Cer(d15:0/24:0) | 978.6994975 | 398.6855 | 3.066457987 | 7.519993567 | 5.814596198 | 9.686671678 | 9.201601932 | 9.813546495 | 2.978559708 | 2.898668712 | 5.125760363 | 2.969741617 | 29.74322107 | 34.79745723 | 32.0228223 | 34.34324025 | 40.17948508 | 31.333385 | 39.98012908 | 35.79614163 | 34.06947885 | 28.06388543 |
| NEG | Hex2Cer(d14:1/26:1) | 988.6837714 | 368.543 | 6.31883493 | 6.142565502 | 6.726127815 | 4.57634909 | 13.56941571 | 7.472498293 | 3.448342408 | 4.205923223 | 8.904962297 | 4.311068563 | 75.2973541 | 98.38950935 | 93.90997375 | 99.29057447 | 104.6680304 | 93.3594248 | 120.8229751 | 102.9789975 | 102.8431484 | 66.78355253 |
| NEG | Hex2Cer(d16:0/26:0) | 1020.735397 | 437.381 | 5.740769172 | 9.699245338 | 6.214508605 | 1.337779047 | 10.94405933 | 6.306071195 | 3.343077538 | 3.665111783 | 5.55356848 | 3.44670291 | 178.0710237 | 233.6416345 | 195.3700783 | 241.2320203 | 208.5532858 | 202.6817233 | 279.5722603 | 259.356116 | 193.405217 | 158.2376942 |
| NEG | PA(18:1/14:1) | 643.4407778 | 141.425 | 2.373577205 | 2.562775892 | 1.523782128 | 1.530356138 | 6.864173538 | 3.798889248 | 1.983763572 | 1.961145523 | 2.104725145 | 1.640790978 | 59.03712837 | 77.64458175 | 81.97996352 | 76.94873673 | 91.52686468 | 79.0226339 | 93.95476722 | 83.7455063 | 85.34054652 | 64.43204408 |
| NEG | PA(18:0/14:0) | 647.4530503 | 141.413 | 3.049844025 | 2.84664755 | 1.815981482 | 1.382216752 | 9.442906462 | 4.039633713 | 1.639154192 | 2.225611257 | 3.006393172 | 1.447292418 | 42.15091125 | 66.59861135 | 67.44511867 | 70.5698501 | 71.3472164 | 69.2547934 | 74.94804498 | 71.88561258 | 65.25595308 | 42.07065813 |
| NEG | PA(16:0/20:0) | 703.5396903 | 389.3805 | 19.75108055 | 9.058474385 | 7.654790607 | 8.057451385 | 37.18308853 | 11.44812732 | 8.566897185 | 8.50079802 | 10.711203 | 7.834184793 | 97.16249148 | 168.3877558 | 188.5070868 | 164.9997378 | 160.4031789 | 189.1764097 | 236.734689 | 178.3757728 | 171.6349778 | 138.7543683 |
| NEG | PA(16:0/22:0) | 731.5769936 | 445.856 | 13.33943681 | 8.044349063 | 10.9724202 | 11.60559508 | 39.07114243 | 11.4295505 | 5.723490805 | 7.138443647 | 14.00347198 | 6.563240567 | 105.8712418 | 162.1864265 | 169.8732907 | 131.189965 | 138.4378889 | 136.1447486 | 160.2032008 | 188.2191075 | 157.3842797 | 104.0949472 |
| NEG | PA(18:0/22:0) | 759.6090749 | 475.4425 | 14.15400713 | 10.4994639 | 13.80524671 | 3.482236853 | 38.15803793 | 15.83125018 | 3.162579652 | 6.583393595 | 14.22625008 | 7.128207303 | 64.23289735 | 71.4886482 | 102.4972574 | 84.1790392 | 98.93830427 | 85.59439767 | 99.95196762 | 95.15954018 | 118.1201906 | 65.59252133 |
| NEG | PI(18:1/16:1) | 833.5121494 | 212.585 | 34.87851513 | 17.80342867 | 44.89882355 | 15.04483135 | 115.2043179 | 55.45806382 | 24.24641373 | 21.40734598 | 28.9994418 | 33.06875662 | 191.1604822 | 325.176574 | 189.7313283 | 238.4934538 | 419.325637 | 277.9448378 | 520.1298375 | 199.0970778 | 318.3343467 | 137.801611 |
| NEG | PI(18:0/18:0) | 865.5652166 | 290.348 | 9.472760765 | 17.55021697 | 15.20975285 | 9.494642133 | 33.0773498 | 18.45943578 | 9.456222023 | 7.232419083 | 9.822014967 | 10.16203974 | 149.3031701 | 236.8621475 | 165.507943 | 206.2931918 | 324.1656438 | 217.7542605 | 378.466627 | 177.7124297 | 244.2139732 | 104.655992 |
| NEG | PI(17:0/20:4) | 871.5272557 | 227.748 | 2.294888202 | 1.184053523 | 1.674508012 | 1.14131152 | 2.606180213 | 1.559146082 | 1.2494191 | 1.038099423 | 1.461315703 | 1.097364427 | 74.01758017 | 123.05022 | 80.00709648 | 95.9266144 | 156.701082 | 110.224801 | 191.0408257 | 84.20767833 | 117.6121299 | 51.4611366 |
| NEG | PI(20:2/18:0) | 889.5725199 | 294.954 | 11.49105364 | 16.71550123 | 14.79447893 | 12.30517783 | 34.12008497 | 18.54032953 | 7.812231318 | 6.84996374 | 7.742100153 | 8.912011947 | 70.10756055 | 98.51772325 | 73.8156188 | 85.77685182 | 126.1366559 | 100.1063198 | 162.4815043 | 78.16510072 | 104.0371029 | 27.82107752 |
| NEG | PI(18:0/20:2) | 889.5663913 | 267.9615 | 5.223941872 | 4.239767228 | 6.088388928 | 3.75710482 | 11.93014372 | 4.951746573 | 1.881773065 | 2.957027868 | 2.431610957 | 3.793713418 | 133.0957969 | 204.9625 | 134.8819611 | 161.844152 | 243.1675175 | 183.683744 | 317.854862 | 144.2534613 | 211.5126128 | 88.22003698 |
| NEG | PI(18:0/22:5) | 911.5580073 | 250.096 | 1.277008895 | 1.770625017 | 1.617974968 | 1.090929122 | 2.675330865 | 1.576147287 | 1.060235865 | 1.160370103 | 1.343924363 | 1.34100278 | 66.9778719 | 94.60497155 | 76.28430855 | 81.9485583 | 118.0607287 | 89.06282602 | 151.2379799 | 75.62328197 | 105.1656915 | 44.9834217 |

Table S2. Identification of significantly different polar lipids between donkey colostrum and bovine colostrum.

| Compounds | *P*-value | FC | log2 FC | VIP | MZ | RT |
| --- | --- | --- | --- | --- | --- | --- |
| PC(16:0/0:0) | 1.43E-14 | 5.5885 | 2.482461104 | 1.06404 | 496.3342889 | 86.524 |
| PC(P-20:0/15:1) | 2.86E-13 | 7.40591 | 2.888677017 | 1.06279 | 758.6155482 | 453.576 |
| PC(P-20:0/18:2) | 1.37E-11 | 7.31663 | 2.871179304 | 1.05225 | 798.6572707 | 423.8045 |
| Cer(d16:1/16:0) | 5.37E-12 | 18.3465 | 4.197432958 | 1.05196 | 510.4835111 | 306.534 |
| Cer(d18:1/16:0) | 1.72E-10 | 20.4465 | 4.353782001 | 1.03442 | 538.5152762 | 354.718 |
| Cer(d16:1/22:0) | 2.65E-11 | 44.508 | 5.475992769 | 1.04821 | 594.5775694 | 439.503 |
| Cer(d15:0/24:1) | 5.68E-11 | 28.3368 | 4.824604943 | 1.03498 | 608.5929817 | 457.842 |
| Cer(d16:1/24:1) | 1.16E-11 | 26.9097 | 4.750054402 | 1.04771 | 620.5932083 | 440.323 |
| Cer(d18:1/22:0) | 7.39E-14 | 38.9327 | 5.282910495 | 1.06392 | 622.6069902 | 474.7575 |
| Cer(d18:1/24:1) | 2.29E-12 | 10.1752 | 3.346985247 | 1.05711 | 648.6236529 | 474.1585 |
| Cer(d18:1/24:0) | 1.17E-12 | 22.7685 | 4.508967344 | 1.05692 | 650.6394814 | 505.753 |
| SM(d14:0/18:1) | 1.12E-09 | 18.162 | 4.182851176 | 1.02216 | 675.5397719 | 261.557 |
| SM(d15:0/18:1) | 3.96E-10 | 18.0063 | 4.170429856 | 1.02024 | 689.5551345 | 285.969 |
| SM(d14:1/20:0) | 5.61E-09 | 30.6058 | 4.935733174 | 1.00168 | 703.571219 | 317.7295 |
| SM(d14:0/20:0) | 3.61E-11 | 15.0576 | 3.912419935 | 1.0488 | 705.5828309 | 435.422 |
| SM(d15:1/20:0) | 3.92E-11 | 31.2047 | 4.963691437 | 1.04993 | 717.581365 | 434.286 |
| SM(d15:0/20:0) | 3.80E-12 | 9.48198 | 3.24518835 | 1.05398 | 719.5958285 | 463.78 |
| SM(d14:1/22:0) | 3.44E-13 | 8.37911 | 3.066797014 | 1.06577 | 731.5966206 | 451.77 |
| SM(d15:1/22:0) | 1.78E-08 | 6.0403 | 2.594620205 | 1.00345 | 745.6046265 | 435.443 |
| SM(d15:0/24:0) | 1.55E-12 | 5.58636 | 2.481908548 | 1.05715 | 775.6599641 | 436.0565 |
| SM(d15:0/26:1) | 3.11E-09 | 6.42171 | 2.682957515 | 1.01342 | 801.6777254 | 525.31 |
| HexCer(d18:1/16:0) | 1.32E-13 | 12.8281 | 3.6812356 | 1.06145 | 700.5683246 | 303.9685 |
| HexCer(d18:1/22:0) | 2.60E-14 | 56.5373 | 5.821131082 | 1.06578 | 784.6627482 | 430.264 |
| HexCer(d17:1/24:0) | 2.00E-09 | 6.37653 | 2.672771547 | 1.01765 | 798.6777178 | 447.488 |
| HexCer(d18:1/24:0) | 2.31E-11 | 8.73869 | 3.127417024 | 1.03816 | 812.6945973 | 464.7275 |
| PE(16:0/0:0) | 1.72E-11 | 7.53935 | 2.914440148 | 1.04696 | 452.2760522 | 82.058 |
| PE(18:1/0:0) | 7.06E-13 | 8.31197 | 3.055190447 | 1.06013 | 478.2920201 | 84.22 |
| PE(18:0/0:0) | 1.63E-15 | 29.3254 | 4.874078882 | 1.07291 | 480.3073236 | 107.398 |
| PE(20:4/0:0) | 2.98E-14 | 27.9351 | 4.80400708 | 1.06517 | 500.2753512 | 67.2065 |
| PE(P-16:0/0:0) | 8.33E-14 | 28.8658 | 4.851289304 | 1.06379 | 436.2813031 | 93.116 |
| PC(24:0/4:0) | 5.69E-14 | 31.6686 | 4.984981185 | 1.06125 | 722.4941674 | 275.5675 |
| PC(12:0/18:1) | 1.22E-09 | 21.0829 | 4.398001421 | 1.00458 | 748.507794 | 281.829 |
| PC(18:1/12:0) | 5.33E-11 | 32.4051 | 5.018148981 | 1.03418 | 748.5157301 | 261.226 |
| PC(14:0/16:0) | 3.22E-13 | 34.6884 | 5.116381393 | 1.06122 | 750.5262917 | 328.872 |
| PC(16:0/14:0) | 1.17E-17 | 16.5996 | 4.053076572 | 1.07432 | 750.5249118 | 284.126 |
| PC(16:0/15:0) | 6.65E-14 | 42.9534 | 5.424700428 | 1.05355 | 764.5397446 | 349.549 |
| PC(14:0/18:2) | 9.93E-10 | 6.77682 | 2.760608452 | 1.0153 | 774.5236398 | 288.4975 |
| PC(14:0/18:1) | 2.66E-12 | 19.5817 | 4.291434114 | 1.05206 | 776.5394196 | 331.2105 |
| PC(16:0/16:0) | 2.17E-12 | 10.1922 | 3.349393588 | 1.04947 | 778.5564887 | 376.866 |
| PC(14:0/20:4) | 4.93E-11 | 16.7544 | 4.066468117 | 1.03253 | 798.5238156 | 274.139 |
| PC(16:0/18:1) | 7.98E-13 | 67.0055 | 6.066207616 | 1.05307 | 804.5727436 | 376.311 |
| PC(18:0/16:0) | 7.77E-13 | 26.2286 | 4.713068898 | 1.05436 | 806.5769933 | 377.3845 |
| PC(17:0/18:1) | 7.82E-11 | 50.0208 | 5.644456226 | 1.03355 | 818.5857672 | 404.9255 |
| PC(16:0/20:5) | 2.38E-09 | 11.3398 | 3.503323291 | 1.01683 | 824.537685 | 286.0245 |
| PC(18:1/18:1) | 1.44E-09 | 6.96241 | 2.799586773 | 1.0204 | 830.5842654 | 470.044 |
| PC(18:0/18:1) | 1.27E-12 | 111.048 | 6.7950396 | 1.04903 | 832.6014436 | 436.502 |
| PC(20:4/18:1) | 7.85E-14 | 126.95 | 6.988116585 | 1.06036 | 852.5685862 | 327.182 |
| PC(20:3/18:1) | 4.10E-13 | 50.145 | 5.648033951 | 1.05637 | 854.5753374 | 326.265 |
| PC(18:0/20:3) | 8.49E-11 | 42.3079 | 5.402855173 | 1.03504 | 856.5991717 | 402.367 |
| PC(18:0/22:5) | 4.34E-10 | 56.9065 | 5.830521545 | 1.01857 | 880.5992827 | 370.3715 |
| PE(16:0/12:0) | 4.86E-14 | 21.913 | 4.453715105 | 1.06199 | 634.4408242 | 257.325 |
| PE(12:0/18:1) | 2.64E-13 | 14.112 | 3.818850561 | 1.05823 | 660.4567471 | 261.721 |
| PE(16:0/14:0) | 1.47E-15 | 14.1626 | 3.824014238 | 1.07006 | 662.4721363 | 305.9265 |
| PE(14:0/18:2) | 1.27E-11 | 7.56427 | 2.91920086 | 1.04348 | 686.471842 | 271.2065 |
| PE(14:0/18:1) | 3.56E-15 | 11.7367 | 3.552954919 | 1.06698 | 688.4881889 | 309.44 |
| PE(16:0/16:0) | 4.91E-15 | 10.386 | 3.376568225 | 1.06541 | 690.4940062 | 309.173 |
| PE(15:0/18:2) | 6.89E-13 | 14.3359 | 3.841560574 | 1.05398 | 700.4876531 | 293.78 |
| PE(16:0/17:1) | 3.36E-15 | 27.3469 | 4.773305396 | 1.0653 | 702.5032661 | 331.9775 |
| PE(16:0/17:0) | 2.13E-15 | 15.8229 | 3.983942134 | 1.06953 | 704.518365 | 366.7955 |
| PE(14:0/20:4) | 1.56E-13 | 42.7394 | 5.41749475 | 1.05957 | 710.4722218 | 261.5095 |
| PE(16:0/18:2) | 8.35E-13 | 6.84844 | 2.775775395 | 1.05529 | 714.5053257 | 318.307 |
| PE(16:0/18:1) | 2.43E-15 | 17.0869 | 4.094818774 | 1.06882 | 716.5209217 | 353.832 |
| PE(18:0/16:0) | 9.51E-15 | 11.9889 | 3.58362739 | 1.06591 | 718.5341011 | 395.334 |
| PE(18:2/17:1) | 2.41E-11 | 7.20613 | 2.849224679 | 1.03971 | 726.5031111 | 302.4355 |
| PE(17:0/18:2) | 1.89E-14 | 16.0852 | 4.007661969 | 1.06585 | 728.5180869 | 339.6315 |
| PE(17:0/18:1) | 1.74E-14 | 12.6978 | 3.666506655 | 1.0645 | 730.5245808 | 339.19 |
| PE(18:0/17:0) | 1.47E-16 | 34.23 | 5.097189387 | 1.06941 | 732.5407215 | 375.945 |
| PE(16:0/20:5) | 1.38E-12 | 66.6913 | 6.059426666 | 1.05541 | 736.486935 | 276.33 |
| PE(18:2/18:2) | 8.34E-10 | 5.04008 | 2.333446633 | 1.01876 | 738.5043828 | 286.9775 |
| PE(18:1/18:2) | 5.89E-12 | 5.46866 | 2.451187369 | 1.0486 | 740.5214859 | 322.8975 |
| PE(18:0/18:2) | 1.00E-11 | 5.35012 | 2.419571251 | 1.04733 | 742.5260922 | 322.065 |
| PE(18:1/18:1) | 3.25E-15 | 17.9054 | 4.162322843 | 1.0685 | 742.5368018 | 359.702 |
| PE(18:0/18:1) | 1.53E-14 | 71.7902 | 6.165715012 | 1.06572 | 744.5511375 | 398.6425 |
| PE(18:0/18:0) | 3.25E-14 | 57.146 | 5.836580614 | 1.06453 | 746.5565988 | 398.577 |
| PE(17:0/20:3) | 1.81E-14 | 9.87566 | 3.303877168 | 1.05405 | 754.5331212 | 346.6705 |
| PE(19:0/18:2) | 2.42E-13 | 10.6736 | 3.415974946 | 1.05896 | 756.5492389 | 381.35 |
| PE(19:0/18:1) | 1.01E-12 | 28.3677 | 4.826177279 | 1.05417 | 758.5650663 | 417.751 |
| PE(18:2/20:4) | 2.71E-12 | 80.4846 | 6.330640858 | 1.04975 | 762.5044188 | 279.129 |
| PE(18:1/20:4) | 1.03E-15 | 95.4682 | 6.576948353 | 1.06889 | 764.5197612 | 312.57 |
| PE(18:0/20:4) | 2.26E-15 | 47.2224 | 5.561399461 | 1.0692 | 766.5258608 | 311.951 |
| PE(18:1/20:3) | 3.12E-15 | 37.7382 | 5.237953707 | 1.0673 | 766.5340559 | 332.36 |
| PE(18:0/20:3) | 1.75E-14 | 17.1709 | 4.101893754 | 1.06387 | 768.5408136 | 333.164 |
| PE(18:1/20:1) | 6.24E-13 | 15.4377 | 3.948385922 | 1.0534 | 770.5580495 | 373.3195 |
| PE(20:0/18:1) | 3.84E-13 | 11.6778 | 3.545696603 | 1.05937 | 772.5801329 | 437.5365 |
| PE(18:1/22:5) | 1.80E-13 | 40.5697 | 5.342330729 | 1.05824 | 790.5353098 | 314.3185 |
| PE(18:0/22:5) | 1.70E-14 | 29.0375 | 4.859845344 | 1.06522 | 792.5494476 | 351.829 |
| PE(22:5/18:0) | 4.99E-11 | 32.7549 | 5.033638839 | 1.0378 | 792.570584 | 404.754 |
| PE(22:4/18:0) | 3.56E-14 | 137.382 | 7.102049182 | 1.06455 | 794.5627944 | 385.7615 |
| HexCer(d18:1/20:0) | 1.20E-13 | 6.31981 | 2.659881186 | 1.06016 | 800.611509 | 473.606 |
| PE(24:0/16:0) | 5.72E-09 | 13.6571 | 3.771579264 | 1.01983 | 802.6344681 | 402.2895 |
| PE(22:6/20:3) | 6.89E-16 | 22.8697 | 4.515365536 | 1.07174 | 812.5360816 | 398.795 |
| PE(24:0/18:0) | 4.07E-11 | 6.86733 | 2.779749292 | 1.03972 | 830.6522923 | 425.4275 |
| HexCer(d19:1/22:0) | 2.29E-14 | 218.985 | 7.774688241 | 1.06423 | 842.6665915 | 441.273 |
| PG(16:1/16:0) | 1.28E-13 | 43.6493 | 5.447886613 | 1.06416 | 719.5023556 | 415.1975 |
| PG(16:1/18:1) | 9.97E-13 | 76.1062 | 6.249942083 | 1.05956 | 745.5184288 | 418.637 |
| PG(16:0/18:1) | 3.55E-10 | 33.2881 | 5.056934622 | 1.02282 | 747.5126625 | 261.6105 |
| PG(18:2/18:1) | 1.39E-12 | 46.985 | 5.554128344 | 1.05922 | 771.5328156 | 421.223 |
| PG(18:1/18:1) | 5.36E-11 | 28.3858 | 4.827097497 | 1.03357 | 773.5271732 | 238.831 |
| PS(18:0/18:2) | 4.28E-09 | 4.06029 | 2.021582773 | 1.00899 | 786.521979 | 351.375 |
| PS(18:0/18:1) | 1.96E-09 | 35.7687 | 5.16062578 | 1.01144 | 788.5397968 | 325.459 |
| PS(18:0/22:4) | 2.55E-11 | 56.3692 | 5.816835188 | 1.03879 | 838.554283 | 314.346 |
| CL(16:0/16:0/18:1/20:0) | 1.03E-14 | 41.3039 | 5.368206105 | 1.06471 | 1434.039861 | 354.5745 |
| CL(18:1/18:1/18:1/18:1) | 1.68E-09 | 8.03089 | 3.005559879 | 1.00854 | 1456.02432 | 321.207 |
| CL(16:0/18:1/18:1/20:0) | 1.53E-15 | 53.5452 | 5.742685347 | 1.06912 | 1460.057317 | 356.714 |
| CL(18:1/18:1/18:1/20:2) | 1.36E-10 | 6.33799 | 2.664025383 | 1.02893 | 1482.039761 | 322.876 |
| CL(18:0/18:0/18:0/20:4) | 3.54E-15 | 39.1181 | 5.289764394 | 1.06639 | 1484.049348 | 354.766 |
| CL(18:1/20:0/18:1/18:1) | 8.36E-15 | 53.0982 | 5.73059105 | 1.0641 | 1486.069345 | 359.277 |
| CL(18:0/18:0/18:0/20:1) | 5.04E-10 | 244.855 | 7.935783846 | 1.01468 | 1490.102535 | 398.3785 |
| CL(18:1/18:1/20:1/20:2) | 3.02E-15 | 50.1416 | 5.647936128 | 1.05937 | 1510.06788 | 358.259 |
| PE(P-16:0/16:1) | 4.35E-11 | 6.19992 | 2.6322496 | 1.0377 | 672.4933481 | 334.887 |
| PE(P-16:0/18:3) | 1.51E-10 | 13.8177 | 3.788445589 | 1.04355 | 696.4925079 | 309.092 |
| PE(P-16:0/18:2) | 1.92E-10 | 5.62341 | 2.491445237 | 1.02994 | 698.5087708 | 344.3285 |
| PE(P-16:0/18:1) | 1.19E-12 | 10.8502 | 3.439649731 | 1.05348 | 700.5245512 | 380.272 |
| PE(P-16:0/20:5) | 1.19E-11 | 55.4511 | 5.793144175 | 1.04304 | 720.4929239 | 299.673 |
| PE(P-16:0/20:4) | 2.98E-15 | 89.3587 | 6.481536292 | 1.06685 | 722.5090467 | 332.339 |
| PE(P-16:0/20:3) | 6.60E-11 | 6.24234 | 2.642086938 | 1.03966 | 724.5239817 | 349.992 |
| PE(P-16:0/22:5) | 4.80E-15 | 39.0305 | 5.286530039 | 1.06572 | 748.5252784 | 335.74 |
| PE(P-18:0/20:4) | 4.59E-14 | 85.1239 | 6.411492345 | 1.06231 | 750.5389298 | 377.4225 |
| PE(P-18:0/20:3) | 9.77E-11 | 50.0627 | 5.645664196 | 1.03141 | 752.5468702 | 377.558 |
| PE(P-18:0/22:5) | 1.46E-12 | 8.11052 | 3.019794415 | 1.05296 | 776.555019 | 378.446 |
| Hex2Cer(d14:0/18:1) | 1.21E-10 | 59.052 | 5.883914017 | 1.02927 | 878.5837819 | 328.056 |
| Hex2Cer(d14:1/20:0) | 7.43E-13 | 116.916 | 6.869328567 | 1.05284 | 906.6098996 | 274.471 |
| Hex2Cer(d14:0/20:0) | 4.15E-12 | 10.3108 | 3.366084369 | 1.04919 | 908.6244457 | 291.282 |
| Hex2Cer(d14:1/22:0) | 2.20E-14 | 23.8604 | 4.576546324 | 1.06374 | 934.6386924 | 320.356 |
| Hex2Cer(d14:0/22:0) | 7.81E-10 | 3.4735 | 1.796390097 | 1.02035 | 936.6534822 | 336.472 |
| Hex2Cer(d14:0/24:1) | 1.09E-14 | 20.3613 | 4.34775777 | 1.06641 | 962.6695824 | 364.015 |
| Hex2Cer(d15:0/24:1) | 3.65E-13 | 39.4666 | 5.302560333 | 1.05895 | 976.6856408 | 384.8965 |
| Hex2Cer(d15:0/24:0) | 6.01E-13 | 5.76091 | 2.526296719 | 1.05314 | 978.6994975 | 398.6855 |
| Hex2Cer(d14:1/26:1) | 5.10E-13 | 14.592 | 3.86710573 | 1.05648 | 988.6837714 | 368.543 |
| Hex2Cer(d14:0/26:1) | 5.90E-14 | 47.8665 | 5.580944415 | 1.06098 | 990.7012125 | 402.9875 |
| Hex2Cer(d15:0/26:1) | 2.57E-15 | 44.7845 | 5.484927594 | 1.06829 | 1004.716191 | 420.392 |
| Hex2Cer(d16:1/26:1) | 2.77E-12 | 20.8861 | 4.384471222 | 1.04891 | 1016.715762 | 401.3015 |
| Hex2Cer(d16:0/26:1) | 9.11E-14 | 51.4645 | 5.685505705 | 1.05769 | 1018.732252 | 437.346 |
| Hex2Cer(d16:0/26:0) | 9.51E-13 | 38.2238 | 5.256399305 | 1.05263 | 1020.735397 | 437.381 |
| Hex2Cer(d17:0/26:1) | 1.25E-15 | 18.2923 | 4.19316458 | 1.06498 | 1032.745287 | 453.525 |
| PA(18:1/14:1) | 1.80E-14 | 30.1258 | 4.912927648 | 1.06633 | 643.4407778 | 141.425 |
| PA(18:0/14:0) | 5.13E-12 | 20.7643 | 4.376033332 | 1.05519 | 647.4530503 | 141.413 |
| PA(16:0/20:0) | 9.11E-11 | 13.1567 | 3.717725769 | 1.03785 | 703.5396903 | 389.3805 |
| PA(18:2/19:0) | 2.12E-11 | 7.15631 | 2.839215884 | 1.03932 | 713.5258036 | 360.255 |
| PA(16:0/22:0) | 2.27E-11 | 11.366 | 3.506652715 | 1.04605 | 731.5769936 | 445.856 |
| PA(18:0/22:0) | 6.33E-10 | 6.97276 | 2.801729825 | 1.02593 | 759.6090749 | 475.4425 |

Table S3. Metabolic pathway identified from the significantly different polar lipids between donkey colostrum and bovine colostrum.

| KEGG pathway | Compound | Raw p | -log10(P) | FDR | Number |
| --- | --- | --- | --- | --- | --- |
| Arachidonic acid metabolism | Phosphatidylcholine | 0.10615 | 0.97408 | 1 | 44 |
| Glycerophospholipid metabolism | Phosphatidylethanolamine; Phosphatidylcholine; Phosphatidylserine | 4.1887e-05 | 4.3779 | 0.003351 | 36 |
| Glycosylphosphatidylinositol (GPI)-anchor biosynthesis | Phosphatidylethanolamine | 0.078082 | 1.1075 | 1 | 32 |
| Sphingolipid metabolism | N-Acylsphingosine | 0.078082 | 1.1075 | 1 | 32 |
| alpha-Linolenic acid metabolism | Phosphatidylcholine | 0.032295 | 1.4909 | 0.86121 | 13 |
| Linoleic acid metabolism | Phosphatidylcholine | 0.012515 | 1.9026 | 0.50062 | 5 |
